# Perturbations in small molecule synthesis uncovers an iron-responsive secondary metabolite network in *Aspergillus fumigatus*

**DOI:** 10.3389/fmicb.2014.00530

**Published:** 2014-10-24

**Authors:** Philipp Wiemann, Beatrix E. Lechner, Joshua A. Baccile, Thomas A. Velk, Wen-Bing Yin, Jin Woo Bok, Suman Pakala, Liliana Losada, William C. Nierman, Frank C. Schroeder, Hubertus Haas, Nancy P. Keller

**Affiliations:** ^1^Department of Medical Microbiology and Immunology, University of Wisconsin-MadisonMadison, WI, USA; ^2^Division of Molecular Biology/Biocenter, Innsbruck Medical UniversityInnsbruck, Austria; ^3^Boyce Thompson Institute and Department of Chemistry and Chemical Biology, Cornell UniversityIthaca, NY, USA; ^4^The J. Craig Venter InstituteRockville, MD, USA; ^5^Department of Bacteriology, University of Wisconsin-MadisonMadison, WI, USA

**Keywords:** *Aspergillus fumigatus*, secondary metabolism, iron, HapX, SreA, hexadehydroastechrome, gene regulation

## Abstract

Iron plays a critical role in survival and virulence of the opportunistic pathogen *Aspergillus fumigatus*. Two transcription factors, the GATA-factor SreA and the bZip-factor HapX oppositely monitor iron homeostasis with HapX activating iron acquisition pathways (e.g., siderophores) and shutting down iron consumptive pathways (and SreA) during iron starvation conditions whereas SreA negatively regulates HapX and corresponding pathways during iron sufficiency. Recently the non-ribosomal peptide, hexadehydroastechrome (HAS; a tryptophan-derived iron (III)-complex), has been found important in *A. fumigatus* virulence. We found that HAS overproduction caused an iron starvation phenotype, from alteration of siderophore pools to regulation of iron homeostasis gene expression including *sreA*. Moreover, we uncovered an iron dependent secondary metabolism network where both SreA and HapX oppositely regulate multiple other secondary metabolites including HAS. This circuitry links iron-acquisition and consumption pathways with secondary metabolism—thus placing HAS as part of a metabolic feedback circuitry designed to balance iron pools in the fungus and presenting iron availability as one environmental trigger of secondary metabolism.

## Introduction

The ubiquitous soil-dwelling filamentous fungus *Aspergillus fumigatus* can cause the life-threating disease invasive aspergillosis in immunocompromised individuals. It is regarded as the most common airborne fungal pathogen of humans (Tekaia and Latge, 2005). *A. fumigatus* has the ability to produce hundreds of bioactive small molecules, so called secondary metabolites (Frisvad et al., 2009). With few exceptions, genes required for their production are generally clustered in fungal genomes, each gene in a specific cluster being subject to common regulatory patterns often triggered by largely unknown environmental cues (Hoffmeister and Keller, 2007; Brakhage, 2013). Some of these secondary metabolites have been shown to contribute to the fungus' virulence (Jahn et al., 2002; Bok et al., 2006; Heinekamp et al., 2012; Berthier et al., 2013; Yin et al., 2013).

One compound recently associated with enhanced virulence is hexadehydroastechrome (HAS), an iron-binding, non-ribosomal peptide-derived molecule (Yin et al., 2013). For the majority of organisms, including fungi, iron is an essential cofactor in several enzymatic reactions and serves as a catalyst in electron transport. However, over-abundance of iron can lead to formation of reactive oxygen species that are highly damaging to cellular components (Halliwell and Gutteridge, 1984). As iron availability is essential for survival of microorganism, the mammalian defense machinery includes iron-withholding systems in order to fight microbial infections (Weinberg, 1999; Weiss, 2002; Fluckinger et al., 2004; Ganz, 2009; Leal et al., 2013). Therefore, controlling iron access during infection is a major determinant of successful microbial infection of the mammalian host. Iron deficiency is also known to serve as a regulatory cue for other virulence determinants in microorganisms (Litwin and Calderwood, 1993; Weinberg, 1999; Oglesby-Sherrouse et al., 2014) and has been shown to induce the production of the virulence factor ribotoxin AspF1 in *A. fumigatus* (Schrettl et al., 2010a).

*A. fumigatus* harbors two high-affinity iron uptake systems, siderophore-assisted iron uptake and reductive iron uptake (Schrettl et al., 2004a; Schrettl and Haas, 2011). Siderophores are non-ribosomal peptide-derived ferric iron chelators (Haas, 2003). *Aspergillus* spp. produces the extracellular siderophores fusarinine C (FC) and triacetylfusarinine C (TAFC) and the intracellular iron storage siderophores ferricrocin (FC) and hydroxyl ferricrocin (HFC) (Oberegger et al., 2001; Schrettl et al., 2007; Wallner et al., 2009). The first devoted step in extra- and intracellular siderophore biosynthesis is catalyzed by the L-ornithine *N*^5^-oxygenase SidA (Schrettl et al., 2004a, 2007). The non-proteinogenic amino acid produced by SidA then bifurcates into two independent non-ribosomal peptide biosynthesis pathways devoted to produce both groups of siderophores (Schrettl et al., 2004a). In contrast to the reductive iron uptake system, siderophore production was shown to be an essential virulence determinant of *A. fumigatus* and other fungal species (Eisendle et al., 2003; Schrettl et al., 2004a, 2007; Oide et al., 2006; Greenshields et al., 2007; Gauthier et al., 2010; Hwang et al., 2012; Leal et al., 2013).

As iron exhibits a Janus-faced role in the physiology of an organism, cellular iron homeostasis is tightly regulated in order to ensure iron availability on one side and prevent toxic iron excess on the other side. In the model organism *A. nidulans*, efficient control of iron homeostasis is executed by the two transcription factors, HapX and SreA that are interconnected in a negative feedback loop (Haas et al., 1999; Hortschansky et al., 2007). HapX is a bZIP protein that is thought to directly sense iron deficiency and execute repression of iron-dependent pathways and *sreA* expression by protein-protein interaction with the CCAAT-binding complex under iron-limiting conditions (Hortschansky et al., 2007). SreA is a DNA-binding GATA-type transcription factor, which represses *hapX* expression and iron acquisition pathways under iron-replete conditions (Haas et al., 1999). Both factors are conserved in *A. fumigatus* and other fungi executing similar roles in iron regulation (Voisard et al., 1993; An et al., 1997; Zhou et al., 1998; Hwang et al., 2008, 2012; Schrettl et al., 2008, 2010a; Gauthier et al., 2010; Wiemann et al., 2012; Leal et al., 2013). In accordance with the requirement of siderophores for establishment of full virulence, deletion of *hapX* was shown to attenuate virulence in *A. fumigatus* and *Fusarium oxysporum* (Schrettl et al., 2010a; Leal et al., 2013; Lopez-Berges et al., 2013).

Considering the importance of iron pools in the fungus, coupled with the iron chelating properties of the virulence factor HAS, we set out to characterize the role of HAS in iron-dependent regulation in *A. fumigatus*. We show that on the one hand HAS and, unexpectedly several other secondary metabolites, are subject to iron-dependent regulation by SreA and HapX and on the other hand, over-production of HAS changes HapX/SreA-target gene expression resulting in perturbations in iron-acquisition and consumption pathways. Taken together, this work implicates iron gradients as important environmental cues regulating secondary metabolite synthesis.

## Materials and methods

### Strains and culture conditions

*A. fumigatus* strains used in this study are listed in Table S1. Strains were maintained as glycerol stocks and activated on solid glucose minimal medium (GMM) at 37°C with appropriate supplements (Shimizu and Keller, 2001). For *pyrG* auxotrophs, the growth medium was supplemented with 5 mM uridine and uracil. Conidia were harvested in 0.01% Tween 80 and enumerated using a hemacytometer. For RNA-seq analysis strains Af293, TWY32.1, and TWY24.121 were inoculated into 50 mL of liquid GMM at 5 x 10^6^ conidia/mL in duplicate and grown at 25°C and 250 rpm for 96 h in ambient light conditions. The mycelium was harvested and lyophilized before RNA extraction. For biomass, siderophore and iron-dependent gene expression analysis, strains CEA17, TWY37.2, TJW109.3, TWY25.5, and TWY28.3 were grown in 50 mL *Aspergillus* minimal media (AMM) according to (Pontecorvo et al., 1953) containing 20 mM glutamine and iron concentrations as indicated in the text according to Schrettl et al. (2008). Strains were grown in triplicates (duplicates for gene expression analysis) for 24 h at 37°C, 250 rpm and ambient light conditions with an initial spore concentration of 5 × 10^6^ conidia/mL. For analysis of secondary metabolites and cluster gene expression, strains ATCC46645, Δ hapX, and Δ sreA were inoculated into 50 mL of GMM containing different iron concentrations as indicated in the text at 5 × 10^6^ conidia/mL. Strains were grown in triplicates (duplicates for gene expression analysis) for 5 days (3 days for gene expression analysis) at 25°C and 250 rpm at ambient light conditions. For growth assays strains TJW55.2, TWY32.1, TWY38.6, TWY24.121, TWY35.1, and TW36.1 indicated amount of conidia were inoculated in 2 μL on solidified (Noble Agar, Difco™, BD, USA) GMM containing indicated iron concentrations and FEC, respectively, and incubated for 3 days at 37°C in the dark.

### DNA isolation, genetic manipulations and southern blot analysis

For DNA isolation, *A. fumigatus* strains were grown for 24 h at 37°C in steady state liquid GMM, supplemented with 1 mM FeSO_4_. DNA isolation was performed as described by Green and Sambrook (2012). The *A. fumigatus* mutant strains were constructed using a double-joint fusion PCR approach (Szewczyk et al., 2006) and all primers used in the study are listed in Table S2. Briefly, approximately 1 kb fragments flanking the targeted deletion region were amplified by PCR from *A. fumigatus* strain Af293 or CEA17 genomic DNA and the *A. parasiticus pyrG* marker gene was amplified from the plasmid pJW24 (Calvo et al., 2004). The three fragments were subjected to fusion PCR to generate deletion cassettes. The *A. parasiticus pyrG* marker gene was amplified from the plasmid pJW24 using the primer pair pyrG_prom_F/pyrG_term_R (Table S2). The primers “gene”-5R and “gene”-3F contain complement sequences to the primers pyrG_prom_F and pyrG_term_R at their 5'-region, respectively (Table S2). The fusion construct was created by PCR containing 5′ and 3′ gene flanks and the *pyrG* gene fragment functioning as templates and primers simultaneously. The final PCR fusion product was amplified using primer pairs “gene”-5F/“gene”-3R and the previously PCR-generated fusion construct as template. Transformation was performed as described by Palmer et al. (2008). For selection of Δ sidA transformants, 1 mM FeSO_4_ was added to the selection media. For multiplex diagnostic PCR, primer pair “gene”-F/“gene”-R were used to identify transformants that lost the respective gene locus and primer pair gpd_int-F/gpd_int-R as internal control (Table S2). Single integration of the transformation construct was confirmed by Southern analysis as described by Green and Sambrook (2012) using P^32^-labeled probes created by amplification of the respective deletion construct using primer pair “gene”-5F/“gene”-3R (Table S2 and Figure S3).

### RNA extraction and northern analysis

Mycelia were harvested by filtration through Miracloth (Calbiochem). Total RNA was extracted with TRIzol reagent (Invitrogen) from freeze-dried mycelia, following the manufacturer's protocol. Northern analysis was performed as described by Green and Sambrook (2012). Probes for northern analysis were constructed at regions internal to the gene of interest using primers listed in Table S2 (“gene”-F/“gene”-R) and labeled with dCTP αP^32^.

### RNA sample preparation and illumina sequencing (RNASeq)

To identify transcriptionally active genes, extracted RNA samples were subject to DNase treatment using the RNeasy kit (Qiagen), and sequencing libraries were generated using the ScriptSeq kit v2 (Epicenter) following manufacturer's directions. All libraries were sequenced (7 sample per lane) using the Illumina HiSeq2000 instrument (www.illumina.com) on a 2 × 100 bp paired-end run. Transcripts were assembled and expression levels were estimated using normalized reads per kilobase per million mapped reads (RPKM) (Mortazavi et al., 2008) as calculated by the TopHat, Bowtie, and Cufflinks packages (Trapnell et al., 2009, 2010, 2012; Langmead et al., 2010) and CLC Genomics Workbench (CLC Bio). After trimming for quality (>Q30), reads were mapped at 90% length, 90% identity and reads that mapped to more than one location in the genome were excluded. A minimum of 4 reads in each condition were required to analyze gene expression, over 88% of genes had over 4 reads in every condition. Differentially expressed genes were determined in duplicate data by identifying genes that were at least two-fold difference using a *T*-test cutoff of *p* < 0.05 (Bullard et al., 2010) and by using SAM with a false discovery rate of 0 as implemented in MeV (Saeed et al., 2006). Replicates of each condition were highly reproducible (*R*^2^ > 0.99) (Dataset S1). For gene enrichment analysis, genes differentially regulated were analyzed for enrichment using the Munich Information Center for Protein Sequences (MIPS) Functional Catalog (FunCat) (Ruepp et al., 2004) at http://www.helmholtz-muenchen.de/en/ibis/resourcesservices/index.html (Dataset S2).

### Siderophore and secondary metabolite analysis

Analysis of siderophores was carried out by reversed phase high performance liquid chromatography (HPLC) as described previously (Oberegger et al., 2001). Moreover, to quantify extracellular or intracellular siderophores, culture supernatants or cellular extracts were saturated with FeSO_4_ and siderophores were extracted with 0.2 volumes of phenol. The phenol phase was separated and subsequent to addition of five volumes of diethylether and 1 volume of water, the siderophore concentration of the aqueous phase was measured photometrically using a molar extinction factor of 2996 M^−1^ cm^−1^ at 440 nm.

For analysis of pseurotin A, fumitremorgin C and fumagillin supernatant of fungal cultures was lyophilized and re-dissolved in 5 mL double destilled (dd) H_2_O 1% (v/v) formic acid (FA). From the water crude, 800 μL were partitioned with 800 μL ethyl acetate for three times. The combined organic layers of each sample were evaporated *in vacuo*, and re-dissolved in 500 μL of 20% (v/v) acetonitrile (ACN) 1% FA (v/v) and 50 μL were examined by HPLC photo diode array (PDA) analysis. The samples were separated on a ZORBAX Eclipse XDB-C18 column (Agilent, 4.6 mm by 150 mm with a 5 μm particle size) by using a binary gradient of 1% (v/v) FA as solvent A and 1% FA in ACN as solvent B using a Flexar Binary Liquid Chromatography (LC) Pump (PerkinElmer) coupled to a Flexar LC Autosampler (Perkin Elmer) and a Flexar PDA Plus Detector (PerkinElmer). The binary gradient started with an isocratic step at 80% A for 2 min followed by a linear gradient to 40% A in 10 min and an additional linear gradient to 100% B in 0.5 min. After each run the column was washed for 5 min using 100% B and was equilibrated for 4 min using 80% A. The flow rate was set to 1.5 mL/min. Identification and relative quantification of secondary metabolites was performed using Chromera Manager (PerkinElmer) by comparison to standards for fumagillin, pseurotin A and fumitremorgin C (Cayman Chemicals, MI, USA) (Dataset S3). Areas under the curve were normalized to dry weights of each sample. For terezine D analysis liquid fungal cultures including fungal tissue and media were frozen using a dry ice acetone bath, and lyophilized. The lyophilized residues were extracted with 75 mL of 10% methanol in ethyl acetate for 3.5 h with vigorously stirring. Extracts were filtered over cotton, evaporated to dryness, and stored in 8 mL vials. Preparation for HPLC-mass spectrometry (MS) Analysis: Crude extracts were suspended in 0.1 mL of methanol and centrifuged to remove insoluble materials, and the supernatant was subjected to HPLC-mass spectrometry analysis using an Agilent 1100 series HPLC connected to a Quattro II low resolution mass-spectrometer (Micromass/Waters) operated in electrospray positive ionization (ESI^+^). Data acquisition and processing for the HPLC-MS was controlled by Waters MassLynx software. An Agilent Zorbax Eclipse XDB-C18 column (4.6 × 250 mm, 5 μm particle diameter) was used with 0.1% acetic acid in 50/50 methanol/acetonitrile (organic phase) and 0.1 % acetic acid in water (aqueous phase) as solvents at a flow rate of 1.0 mL/min. A solvent gradient scheme was used, starting at 5% organic for 3 min, followed by a linear increase to 100% organic over 35 min, holding at 100% organic for 15 min, then decreasing back to 5% organic for 1 min and holding at 5% organic for the final 6 min, a total of 1 h. Relative abundance of terezine D was determined by integrating their extracted ion chromatograms using MassLynx software (Dataset S3).

### Statistical analysis

Statistical analysis was performed by using GraphPad Prism software using analysis of variance (ANOVA) followed by Tukey's test to show significant differences.

## Results

### HAS does not contribute to siderophore-assisted iron uptake

Since HAS was shown to chelate ferric iron, similar to the intra- and extra-cellular siderophores produced by *A. fumigatus*, we investigated whether HAS could restore growth defects in an *A. fumigatus sidA* deletion mutant deficient of siderophore production. Deletion mutants of *sidA* in *A. fumigatus* were previously shown to exhibit strong growth defects under iron deficient conditions and to be avirulent in animal models of invasive aspergillosis (Schrettl et al., 2004a, 2007; Hissen et al., 2005; Knox et al., 2014). Single (Δ*sidA*) and double (OE::*has*A/ΔsidA) mutants of *sidA* with or without an overexpression *hasA* allele were created as described in Material and Methods (Figure S1). HasA is the *has* cluster transcription factor positively activating other members of the cluster and HAS biosynthesis (Yin et al., 2013).

**Figure 1:**
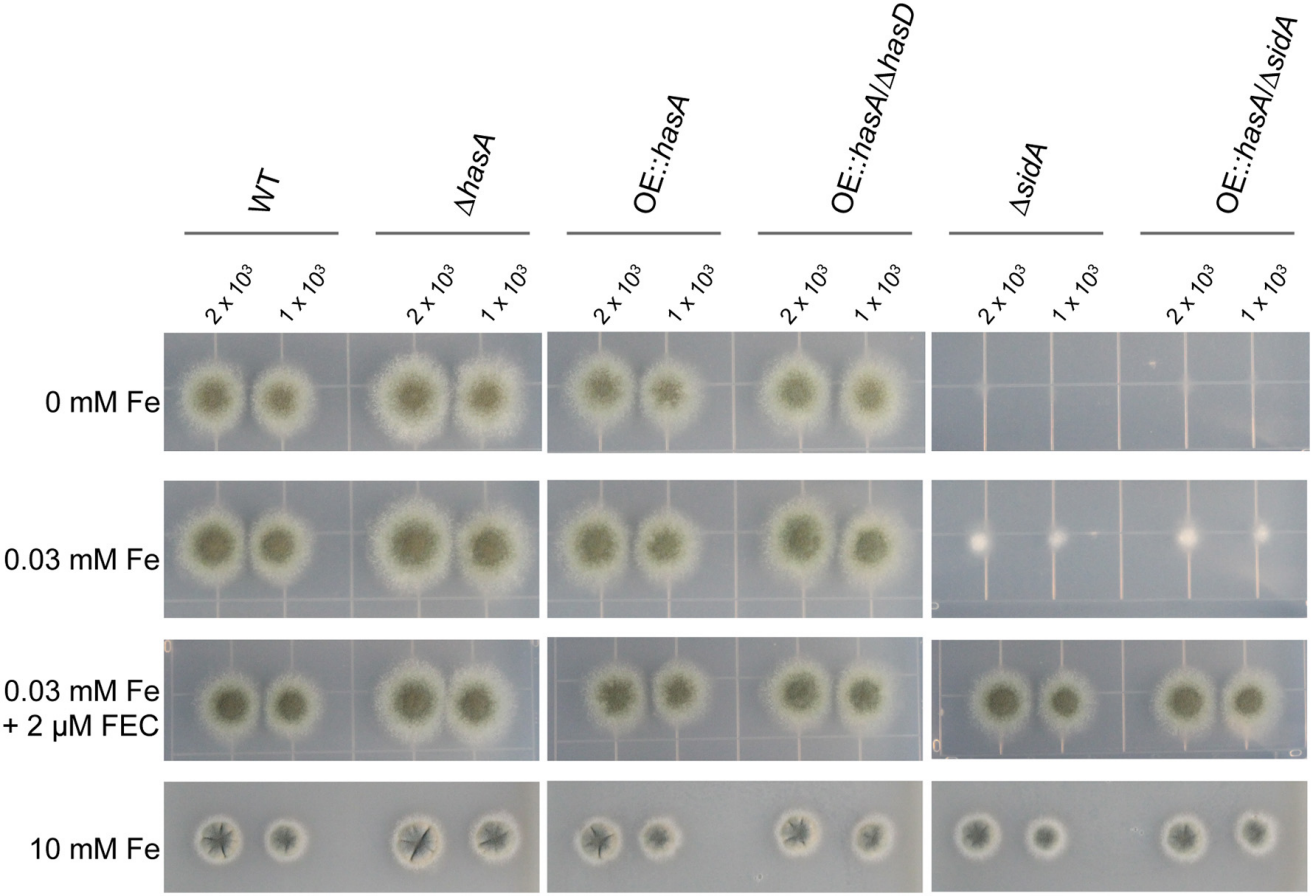
**Growth phenotypes of the wild type, Δ*hasA*, OE::*hasA*, OE::*hasA*/Δ*hasD*, Δ*sidA*, and OE::*hasA*/Δ*sidA* strains on different iron conditions using solidified media**. Indicated amount of spores were spotted on solidified GMM with various iron concentrations and 2 μM ferrichrome (FEC) as indicated. Strains were grown for 3 days at 37°C in the dark.

Confirming previous results (Schrettl et al., 2004a), *sidA* deletion mutants showed significant growth defects under iron depleted conditions compared to the wild type (WT), the *hasA* deletion mutant (Δ*hasA*), the overexpression *hasA* mutant (OE::*hasA*), as well as a mutant overexpressing *hasA* in a *hasD* deletion background (OE::*hasA/*Δ*hasD*) where *hasD* encodes the non-ribosomal peptide synthetase required for HAS biosynthesis (Figure 1). We found that OE::*hasA* in the Δ*sidA* background did not rescue growth (Figure 1), thereby ruling out the hypothesis that HAS could function as an extra-cellular siderophore assisting in iron uptake. Growth of Δ*sidA* and OE::*hasA*/Δ*sidA* could be restored to wild-type like levels when 2 μM of the *Schizosaccharomyces pombe* siderophore ferrichrome (Schrettl et al., 2004b) was supplemented to iron deplete conditions. Under extreme iron excess (10 mM), growth rates of the WT and the OE::*hasA* strains were decreased to similar levels (Figure 3), indicating that HAS, similar to FC and HFC, does not participate in iron detoxification in contrast to the vacuolar iron transporter CccA (Gsaller et al., 2012) (Figure 1).

### HAS and astechrome bind iron *in vivo*

**Figure 2:**
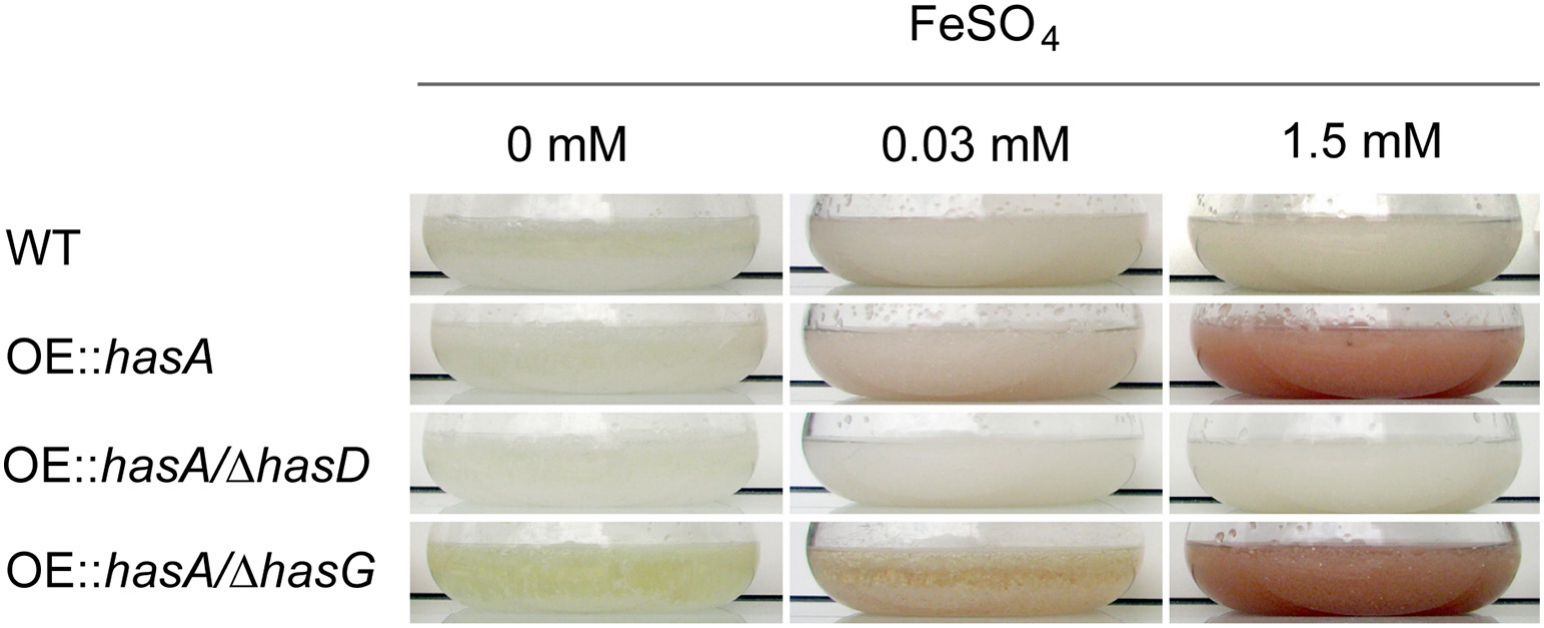
**Growth phenotypes of wild type, OE::*hasA*, OE::*hasA*/Δ*hasD*, and OE::*hasA*/Δ*hasG* strains in different liquid iron conditions**. Indicated strains were incubated in AMM supplemented with indicated iron concentrations for 1 day at 37°C at 200 rpm.

In order to confirm the ability of HAS and its precursor astechrome to bind iron as reported previously (Arai et al., 1981; Yin et al., 2013), we cultivated the WT, OE::*hasA*, OE::*hasA*/Δ*hasD*, and OE::*hasA*/Δ*hasG* strains under increasing iron conditions. HasG is a FAD binding protein required to convert astechrome to HAS and both compounds chelate iron (Arai et al., 1981; Yin et al., 2013). OE::*hasA* and OE::*hasA*/Δ*hasG* exhibited a reddish mycelial coloration particularly during iron excess in contrast to the wild type and OE::*hasA*/Δ*hasD* (Figure 2).

### Overproduction of HAS causes growth defects and increased siderophore production under iron-replete conditions

**Figure 3:**
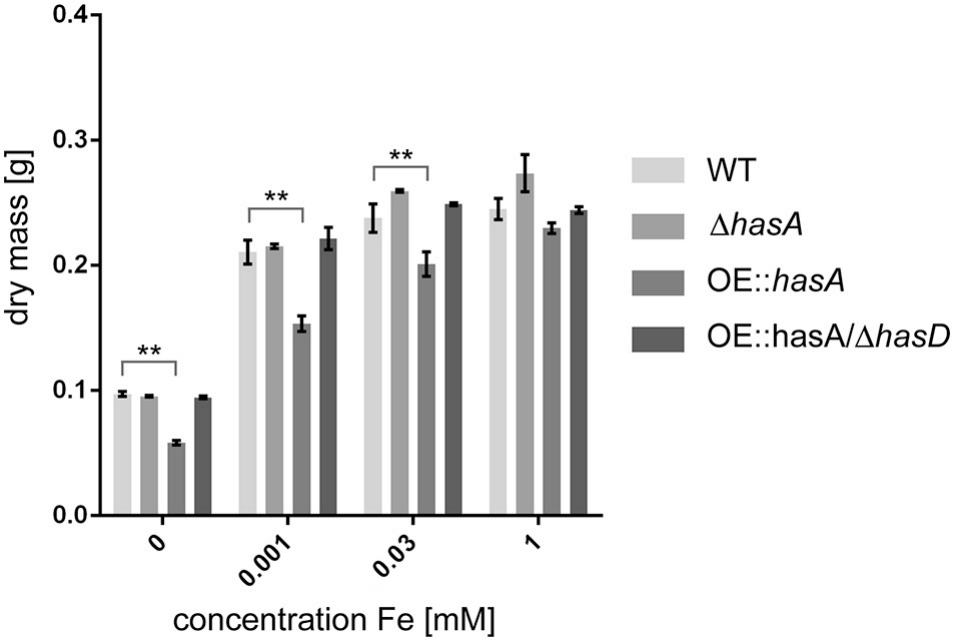
**Mycelial dry weights of wild type, Δ*hasA*, OE::*hasA* and OE::*hasA*/Δ*hasG* under different iron conditions**. Indicated strains were grown in liquid 50 mL AMM for 1 day at 37°C at 250 rpm. Harvested mycelia was lyophilized before weighing. Error bars indicate standard deviations for triplicates of each strain. Asterisks indicate significance as calculated by Tukey's test following ANOVA: ^**^p < 0.05.

Since HAS could be excluded to function as an extracellular siderophore, we investigated if it could function in iron detoxification under iron-replete and iron excess conditions. Therefore, we assessed biomass of the WT, Δ*hasA*, OE::*hasA*, and OE::*hasA*/Δ*hasD* strains. During iron-replete (30 μM), iron deficient (1 μM) and no iron (0 μM) conditions OE::*hasA* showed decreased biomass production compared to the other strains tested (Figure 3). In case of OE::*hasA* this growth defect could be rescued by addition of 1 mM iron to the media (Figure 3). Taken together these data suggest that HAS causes growth deficiency due to its iron-chelating ability.

**Figure 4:**
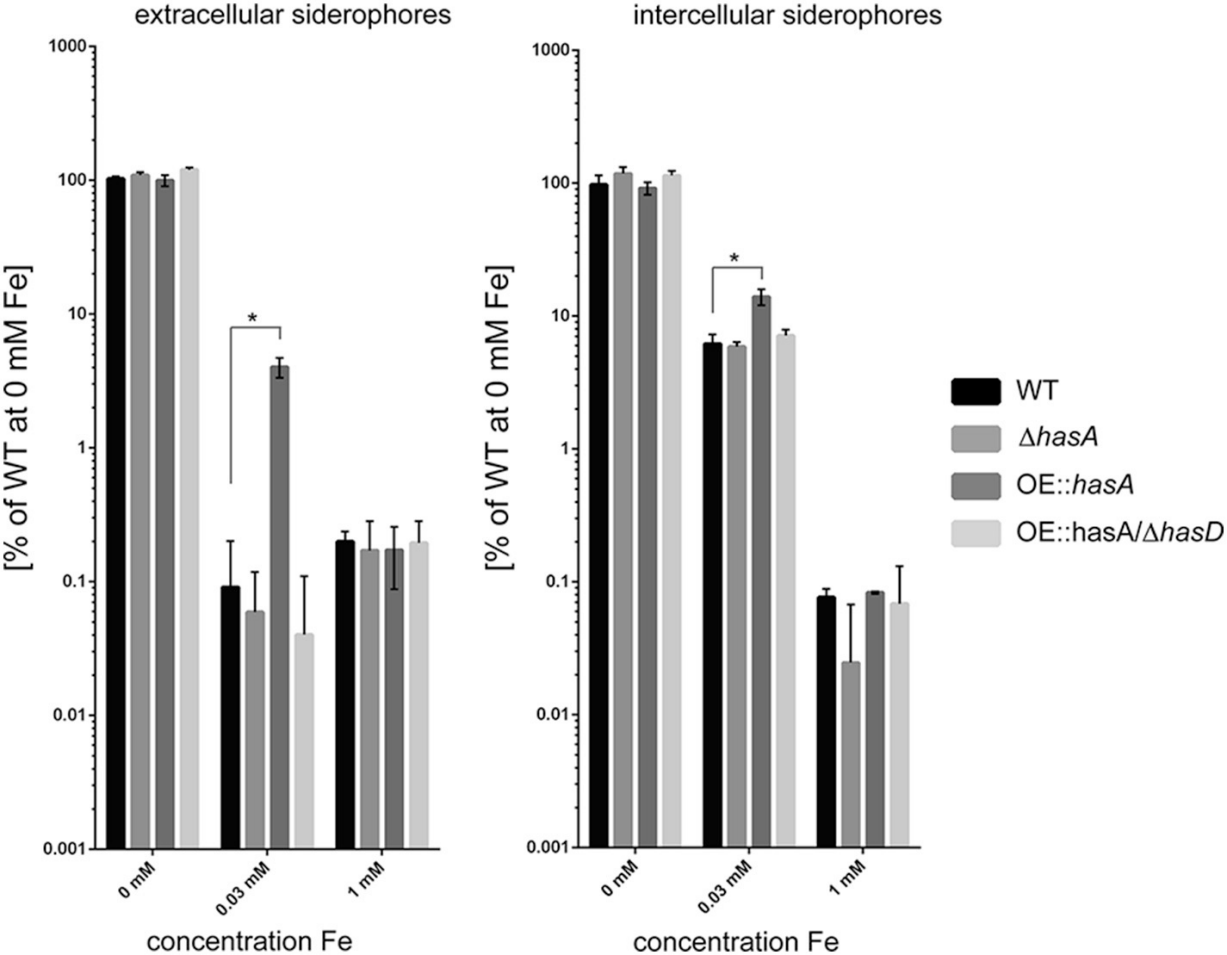
**Production of extra and intracellular siderophores in the wild type, Δ*hasA*, OE::*hasA*, and OE::*hasA*/Δ*hasD* strains in different iron conditions**. Indicated strains were grown in liquid AMM supplemented with indicated iron concentrations at 37°C for 1 day at 200 rpm. Siderophore concentration was measured as described in Material and Methods and normalized to the amount produced by the WT and plotted in log scale. Error bars indicate standard deviations for triplicates of each strain. Asterisks indicate significance as calculated by Tukey's test following ANOVA: ^*^p < 0.0005.

In line with the observed biomass reduction, production of intra- and extra-cellular siderophores was increased under iron-replete conditions (30 μM) in strains overproducing HAS (OE::*hasA*) compared to the WT or OE::*hasA*/Δ*hasD* (Figure 4). At iron-replete conditions (30 μM iron), OE::*hasA* produces more extracellular and intracellular siderophores than WT or the other *has* mutants thus supporting the hypothesis that HAS causes the fungus to experience iron-deficiency in this condition. However, under iron starvation (0 μM) no increase in siderophore production could be observed in OE::*hasA* compared to the WT, most likely due to the fact that siderophore production is already fully induced under those conditions in all strains. In iron excess conditions, siderophore production is similar across all strains (Figure 4).

### HAS and astechrome overproduction increases expression of genes involved in iron acquisition and represses genes involved in iron-dependent pathways

**Figure 5:**
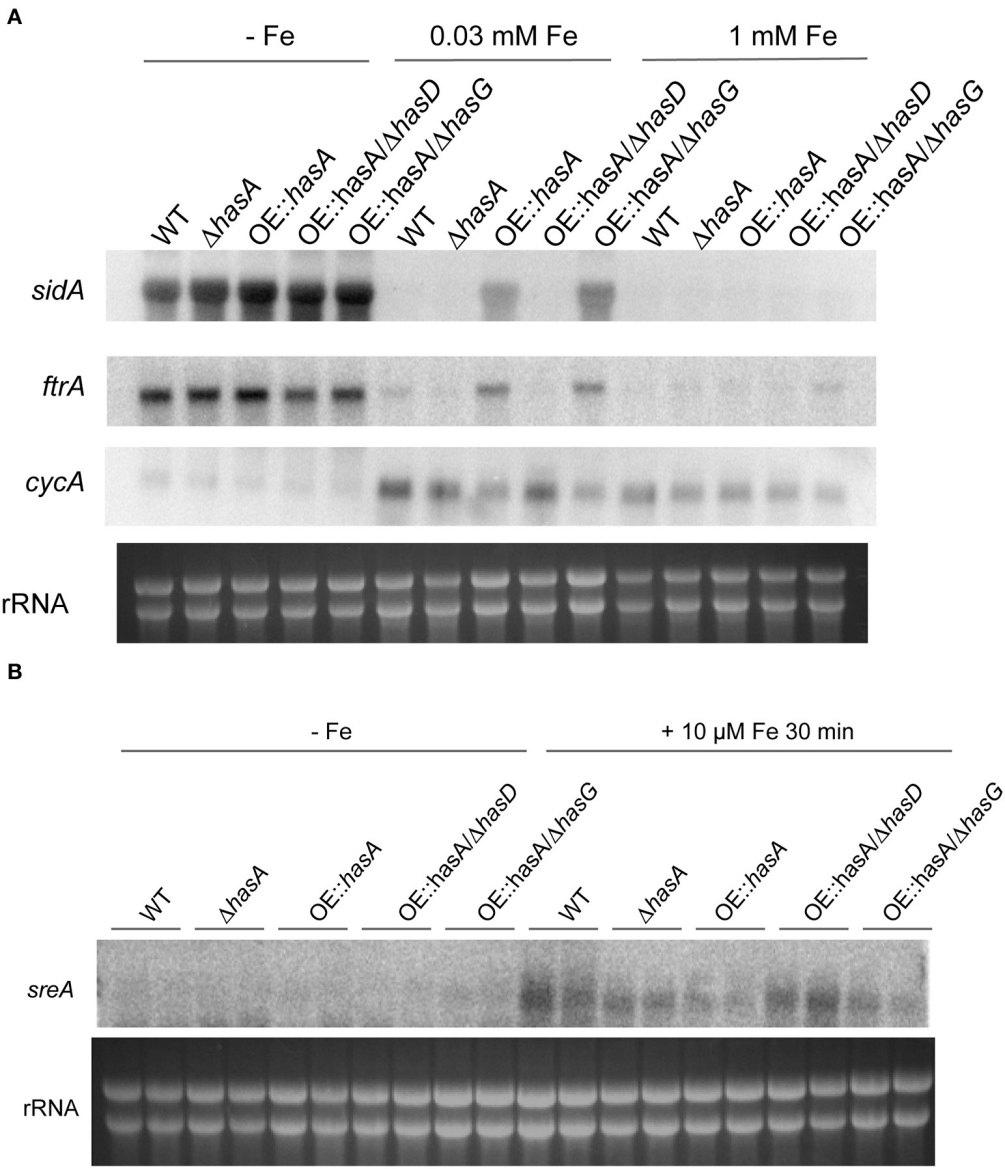
**Expression analysis for select HAS target genes for the wild type, Δ*hasA*, OE::*hasA*, OE::*hasA*/Δ*hasD*, and OE::*hasA*/Δ*hasG* under different iron conditions**. **(A)** Strains were grown for 24 h as described in Material and Methods under the indicated iron conditions. **(B)** Strains were grown for 24 h in −Fe media as described in Material and Methods. One set of cultures was supplemented with 10 μM FeSO_4_ for 30 min before harvesting. Northern blot hybridization of P^32^ labeled DNA probes to indicated target genes. Ethidium bromide-stained rRNA visualized as control.

In *A. fumigatus*, iron starvation induces the expression of genes involved in siderophore biosynthesis (e.g., *sidA*) and reductive iron uptake (e.g., *ftrA*). Moreover, iron starvation represses genes involved in iron dependent pathways such as *cycA* (Schrettl et al., 2010a). During iron-replete conditions (30 μM), OE::*hasA* and OE::*hasA*/Δ*hasG* increase expression of *sidA* and *ftrA* but repress *cycA* expression compared to the WT, Δ*hasA* and OE::*hasA*/Δ*hasD*, respectively (Figure 5A). These data indicate that HAS or astechrome accumulation causes iron starvation, which is in agreement with the reduced biomass formation and increased siderophore production under this condition (Figures 3, 4). Since expression of genes involved in iron acquisition and iron-utilization are repressed by SreA and HapX, respectively, in *A. fumigatus* (Schrettl et al., 2008, 2010a), we tested expression of *sreA* in iron deplete conditions and conditions exposed to iron sufficiency for a short time. As previously described (Schrettl et al., 2008), shifting to iron-replete conditions caused induction of *sreA* expression compared to iron-deplete conditions in the WT. This induction is less pronounced in OE::*hasA* and OE::*hasA*/Δ*hasG* compared to the other strains tested (Figure 5B). These data are in line with the growth and siderophore production data indicating that HAS overexpression decreases iron bioavailability.

### HAS and other secondary metabolites are controlled by ambient iron conditions in a HapX- and SreA-dependent manner

**Figure 6:**
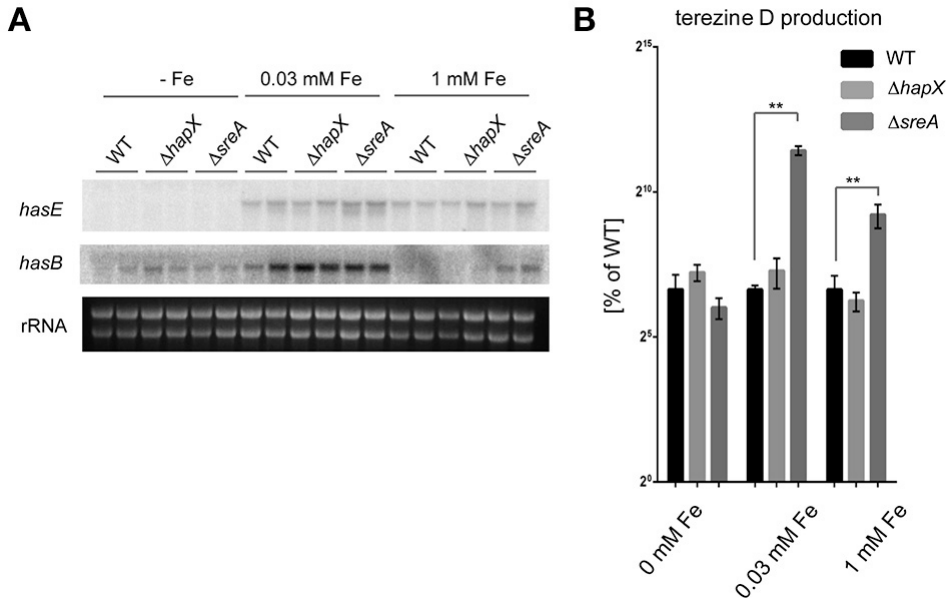
**Analysis of HAS biosynthesis in the wild type, Δ*hapX* and Δ*sreA* mutants under different iron conditions. (A)** Strains were grown for 3 days at 25°C at 250 rpm in GMM supplemented with indicated iron concentrations. Northern blot hybridization of P^32^ labeled DNA probes to indicated target genes. Ethidium bromide-stained rRNA visualized as control. **(B)** Strains were grown at 25°C for 5 days at 250 rpm in GMM supplemented with indicated iron concentrations. Terezine D was measured as described in Material and Methods and plotted as percent of the amount produced in the wild type at 0, 0.03 and 1 mM Fe, respectively, in log scale. Error bars indicate standard deviations for triplicates of each strain. Asterisks indicate significance as calculated by Tukey's test following ANOVA: ^**^p < 0.0005.

As our cumulative data demonstrate a role of HAS in iron homeostasis of *A. fumigatus* we were curious if the *has* gene cluster itself is subject to HapX- or SreA-mediated iron-dependent expression. Therefore, we grew the WT, Δ*hapX*, and Δ*sreA* under iron deplete (0 μM), replete (30 μM) and excess (1 mM) conditions and assessed HAS production and gene expression by northern blot analysis. Expression of select *has* genes was repressed by iron depleted conditions in a HapX-independent manner (Figure 6A). Gene expression of *hasB* and concomitant terezine D production, the first stable intermediate of the HAS pathway, was induced by medium-high iron concentrations and SreA-deficiency, which increases the cellular iron accumulation (Figures 6A,B).

**Figure 7:**
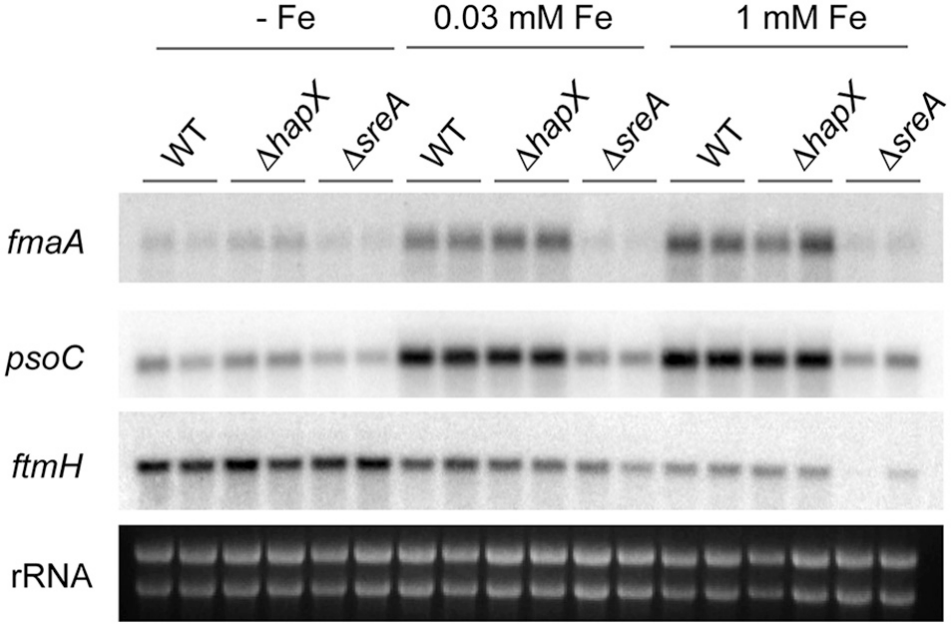
**Expression analysis for select secondary metabolite cluster genes for the wild type, Δ*hapX* and Δ*sreA* mutants under different iron conditions**. Strains were grown for 3 days at 25°C for 3 days at 250 rpm. Northern blot hybridization of P^32^ labeled DNA probes to indicated target genes. Ethidium bromide-stained rRNA visualized as control.

**Figure 8:**
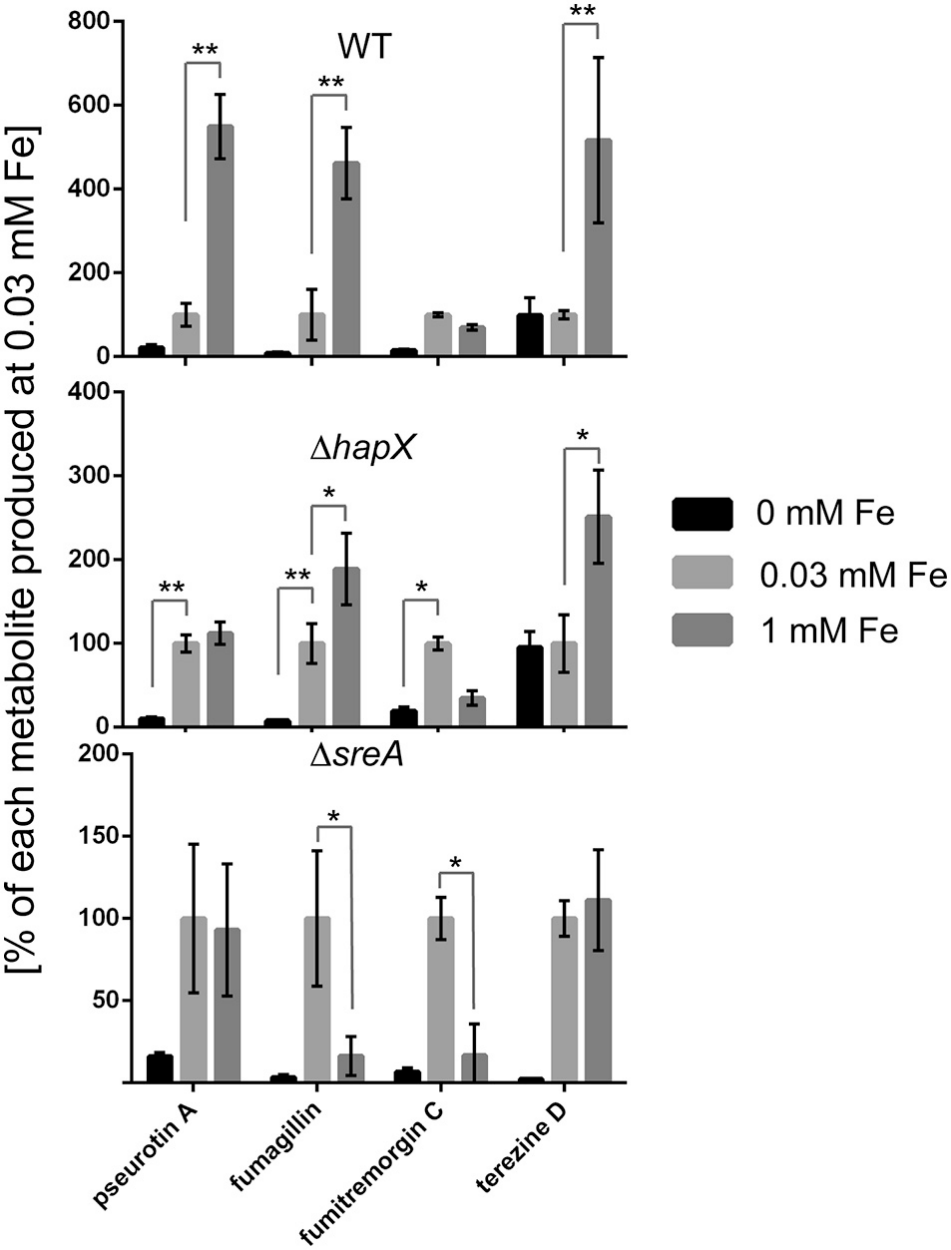
**Secondary metabolite analysis of wild type, Δ*hapX* and Δ*sreA* mutants in an iron-dependent manner normalized to iron replete conditions**. The strains were grown for 5 days in GMM supplemented with indicated concentrations of iron for 5 days at 25°C. Secondary metabolites were analyzed as described in material and Methods and plotted as percent of the amount produced at 0.03 mM for each metabolite in each strain individually. Error bars indicate standard deviations for triplicates of each strain. Asterisks indicate significance as calculated by Tukey's test following ANOVA: ^**^p < 0.0005 and ^*^p < 0.001.

Since we observed an iron-dependent expression of the *has* cluster and HAS production, we investigated expression of select genes from the gene clusters responsible for fumitremorgin C, pseurotin A and fumagillin production, as these secondary metabolites are known to be produced under the same wild-type conditions and therefore convenient to measure (Wiemann et al., 2013). Similar to the observed regulation for the *has* cluster, select genes from the *pso* and *fma* supercluster (Wiemann et al., 2013) were co-activated followed by increased product formation with increasing iron concentration in the WT (Figures 7, 8).

**Figure 9:**
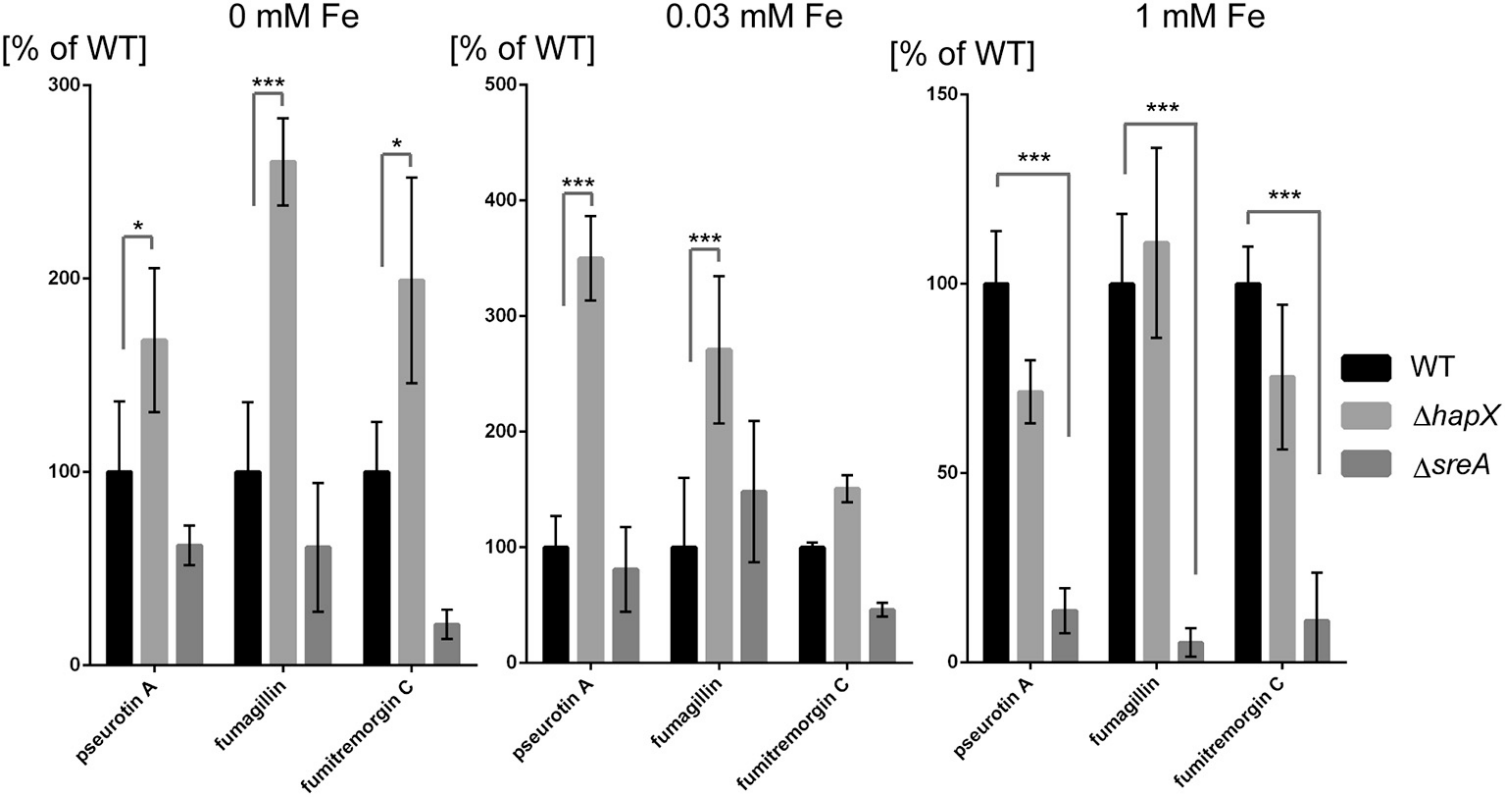
**Secondary metabolite analysis of wild type, Δ*hapX* and Δ*sreA* mutants in an iron-dependent manner normalized to the wild type**. The strains were grown for 5 days in GMM supplemented with indicated concentrations of iron for 5 days at 25°C. Secondary metabolites were analyzed as described in Material and Methods and plotted as percent of the wild-type amount produced by Δ*hapX* and Δ*sreA*, respectively, for each iron condition. Error bars indicate standard deviations for triplicates of each strain. Asterisks indicate significance as calculated by Tukey's test following ANOVA: ^***^p < 0.0001 and ^*^p < 0.05.

Both SreA and HapX loss perturbed metabolite production, reflected in transcriptional regulation in the *sreA* but not *hapX* mutant. In contrast to SreA negative regulation of HAS, fumagillin and pseurotin A production and gene expression was decreased in the Δ*sreA* mutant with increasing iron concentrations (Figures 7, 8). Production of fumagillin and pseurotin A, as well as fumitremorgin, was greatly reduced in Δ*sreA* in high-iron conditions (Figure 9). All four secondary metabolites were differentially produced in the Δ*hapX* mutant depending on iron concentration (Figure 8) with production of fumagillin and pseurotin significantly increased in Δ*hapX* compared to the WT under no-iron and iron-replete conditions (Figure 9).

A curious observation was made for fumitremorgin C where its production followed much of the same trend as fumagillin and pseurotin A in both Δ*sreA* Δ*hapX* mutants (Figures 8, 9) but, unlike fumagillin and pseurotin, stayed unaffected by increasing iron concentration in WT (Figure 8). Interestingly—and in contrast to fumagillin and pseurotin A cluster genes - expression of *ftmH* was highest under iron-deficient conditions, despite the lack of product formation under those conditions (Figure 7). Notably, fumitremorgin C production is significantly increased in the Δ*hapX* mutant compared to the WT under no-iron but not iron excess conditions, indicating that HapX functions as a repressor under these conditions (Figure 9).

### HAS has global influence on genes involved in secondary metabolism and iron acquisition and consumption

**Figure 10:**
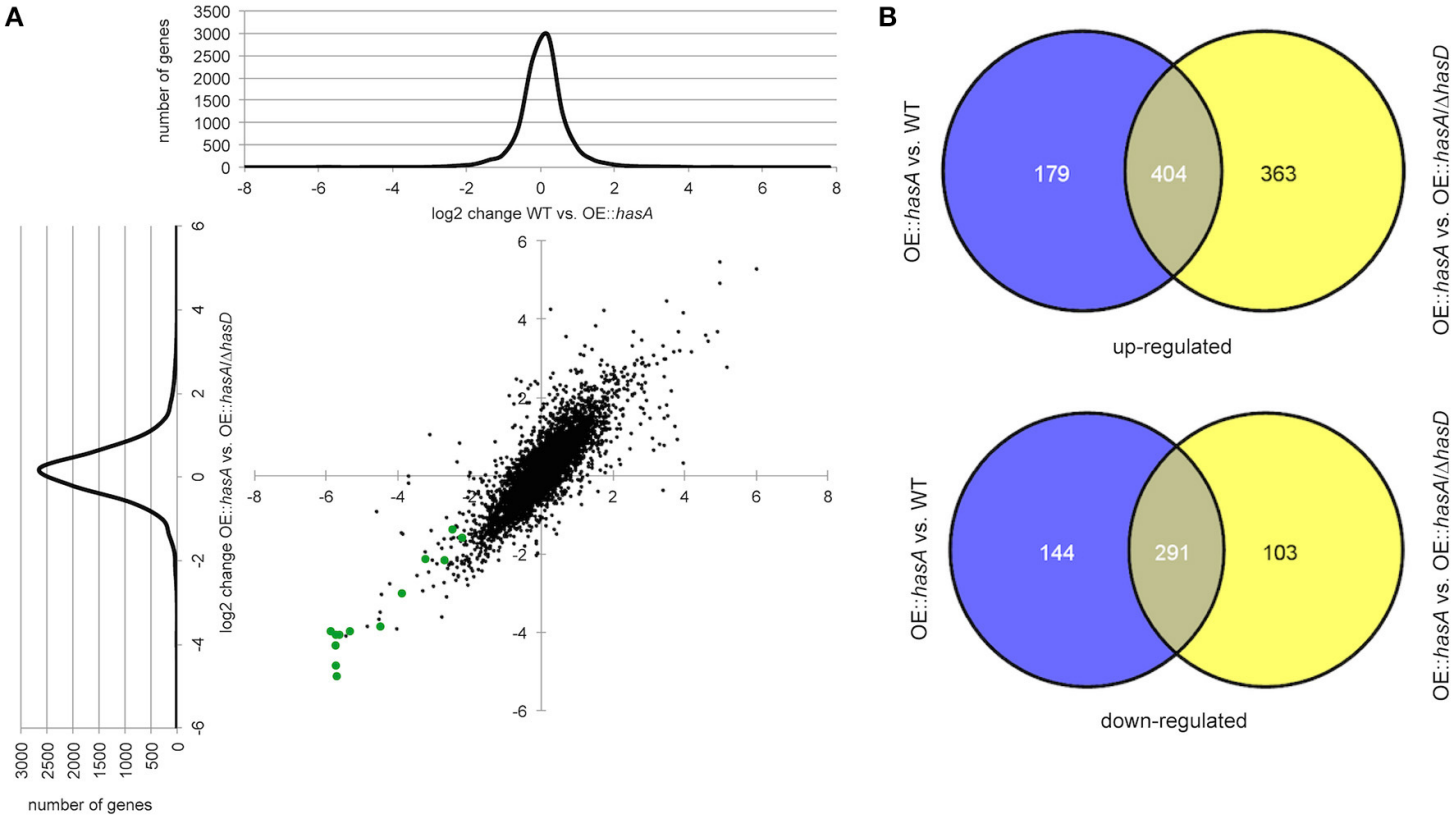
**RNA seq data analysis of gene sets differentially regulated comparing OE::*hasA* to WT and OW::*hasA* to OE::*hasA*/Δ*hasD*. (A)** log(2)-log(2) scatter plot and distribution of genes comparing OE::*hasA* to WT and OW::*hasA* to OE::*hasA*/Δ*hasD*. Genes from the gliotoxin gene cluster are represented in green. **(B)** Number of genes at least two-fold differentially regulated comparing the indicated mutants in Venn diagrams. Numbers in overlapping areas resemble common set of genes in the indicated comparison.

The above data strongly suggested HAS to be a player in an iron acquisition and consumption circuitry involving secondary metabolite production. In order to more thoroughly interrogate these hypotheses, we performed RNA-seq analysis comparing overexpression *hasA* (e.g., OE::*hasA* = high HAS) to WT (low HAS) or to OE::*hasA*/Δ*hasD* (no HAS). Compared to the WT, 583 and 435 genes were up- and down-regulated in the OE::*hasA* strain, respectively. When comparing the OE::*hasA* strain to the strain OE::*hasA*/Δ*hasD*, 767 were up- and 394 genes were down-regulated in the strain able to produce HAS (Figure 10; Dataset S1). A common set of 404 and 291 genes to up- and down-regulated, respectively, was identified between the two comparisons (Figure 10; Dataset S1). Enrichment analysis of these common gene sets of the OE::*hasA* strain compared to both the WT and the OE::*hasA*/Δ*hasD* strains, shows that “secondary metabolism” is a category to be both positively and negatively affected (Figures S1, S2; Dataset S2). Analyzing genes from this category more closely, based on characterized and predicted (Khaldi et al., 2010; Medema et al., 2011; Inglis et al., 2013) gene clusters, provided an accurate view on the genes affected. As expected, genes belonging to the HAS cluster were found to be up-regulated (Table 1).

**Table 1:** **Secondary metabolite cluster genes differentially regulated comparing OE::*hasA* vs. WT and OE::*hasA* vs. OE::*hasA*/Δ*hasD***.

**AFUA ID**	**Gene cluster (number of known genes)**		**log(2) value**	
		
	**Gene name**	**Product**	**OE::*hasA* vs. WT**	**OE::*hasA* vs. OE::*hasA*/Δ*hasD***
**UNCHARACTERIZED PKS CLUSTER**				
AFUA_1G17690	FAD-oxidoreductase	Unknown	1.86	1.70
AFUA_1G17740^*^	PKS		0.56	1.27
**FUMIGACLAVINE (11 GENES)**				
AFUA_2G17980^*^	*fgaPT1*/*easL*	Fumigaclavine	0.13	1.04
AFUA_2G18000^*^	*fgaDH*/*easD*		0.43	1.31
AFUA_2G18010^*^	*fgaP450-2*/*easM*		0.06	1.75
**UNCHARACTERIZED PKS CLUSTER**				
AFUA_3G01400	Transporter	Unknown	2.15	2.34
AFUA_3G01460	Hydroxylase		1.49	2.30
AFUA_3G01500	Hypothetical protein		1.17	1.79
AFUA_3G01560	Permease		1.14	1.42
**UNCHARACTERIZED NRPS-LIKE**				
AFUA_3G02670	NRPS-like	Unknown	2.02	2.59
AFUA_3G02680	Transcription factor		1.47	1.25
AFUA_3G02685	Hypothetical protein		1.21	1.39
AFUA_3G02690	Hypothetical protein		1.74	1.31
AFUA_3G02760	Transporter		1.65	1.61
**HEXADEHYDROASTECHROME (HAS) (8 GENES)**				
AFUA_3G12890	*hasA*	HAS	3.44	1.46
AFUA_3G12900	*hasB*		3.83	3.67
AFUA_3G12910	*hasC*		3.73	1.25
AFUA_3G12920	*hasD*		3.55	1.55
AFUA_3G12930	*hasE*		2.76	1.75
AFUA_3G12940^*^	*hasF*		0.96	1.01
AFUA_3G12950^**^	*hasG*		3.78	0.93
AFUA_3G12960^**^	*hasH*		3.31	0.82
**UNCHARACTERIZED NRPS CLUSTER**				
AFUA_3G13730^*^	*nrps6*/*pesG*	Unknown	0.08	1.25
**UNCHARACTERIZED PKS CLUSTER**				
AFUA_3G14700^*^	PKS	Unknown	0.23	2.07
AFUA_3G14710^*^	Dioxygenase		0.26	2.51
AFUA_3G14740^*^	Hypothetical protein		0.91	1.19
AFUA_3G14750	Transcription factor		1.11	1.26
AFUA_3G14760^*^	P450		0.44	1.47
AFUA_3G14770^*^	NAD(P)^+^-binding protein		−0.18	2.40
**UNCHARACTERIZED PKS CLUSTER**				
AFUA_4G14510	Methyltransferase	Unknown	5.18	2.77
AFUA_4G14520	NAD(P)^+^-binding protein		2.13	2.16
AFUA_4G14530	Glutathione *S*-transferase		1.18	1.49
AFUA_4G14570	Metallo beta-lactamse		2.86	1.60
AFUA_4G14580	*O*-methyltransferase		1.44	1.52
**UNCHARACTERIZED NRPS-LIKE CLUSTER**				
AFUA_5G09960	GPI-anchored protein	Unknown	3.49	1.797
AFUA_5G10040	Transcription factor		1.52	1.52
AFUA_5G10090	Methyltransferase		4.66	3.45
AFUA_5G10160	Nmr-like protein		1.02	1.47
AFUA_5G10180	Monooxygenase		1.32	1.53
AFUA_5G10200	Oxidoreductase		3.97	4.17
AFUA_5G10210	Hypothetical protein		4.99	4.93
AFUA_5G10220	Dehydrogenase		6.00	5.29
**Pes1/PesB CLUSTER**				
AFUA_1G10370	Transporter	Unknown	−1.69	−1.87
**UNCHARACTERIZED PKS CLUSTER**				
AFUA_3G02570	PKS	Unknown	−1.04	−1.38
**UNCHARACTERIZED NRPS CLUSTER**				
AFUA_6G09610	nrps9/*pesJ*	Unknown	−5.45	−4.09
**GLIOTOXIN CLUSTER (13)**				
AFUA_6G09630	*gliZ*	Gliotoxin	−4.49	−3.85
AFUA_6G09640	*gliI*		−5.72	−4.07
AFUA_6G09650	*gliJ*		−5.62	−4.05
AFUA_6G09660	*gliP*		−5.34	−3.98
AFUA_6G09670	*gliC*		−3.89	−2.99
AFUA_6G09680	*gliM*		−5.72	−4.86
AFUA_6G09690	*gliG*		−5.74	−4.34
AFUA_6G09700	*gliK*		−5.87	−3.97
AFUA_6G09710	*gliA*		−5.70	−5.12
AFUA_6G09720	*gliN*		−3.23	−2.14
AFUA_6G09730	*gliF*		−2.21	−1.58
AFUA_6G09740	*gliT*		−2.70	−2.17
AFUA_6G09645	*gliH*		−2.48	−1.38
**PYRIPYROPENE CLUSTER (9)**				
AFUA_6G13980	*pyr6*	Pyripyropene	−1.60	−1.39
AFUA_6G13990	*pyr7*		−1.42	−1.03
AFUA_6G13400	*pyr8*		−2.26	−1.67
**FUMITREMORGIN CLUSTER (8)**				
AFUA_8G00170	*ftmA*	Fumitremorgin	−2.15	−2.44
AFUA_8G00190	*ftmC*		−1.43	−1.39
AFUA_8G00200	*ftmD*		−1.18	−1.24
AFUA_8G00210	*ftmB*		−1.46	−1.21
AFUA_8G00220	*ftmE*		−1.91	−1.57
AFUA_8G00240	*ftmG*		−2.58	−2.27
AFUA_8G00250	*ftmH*		−3.00	−2.82

* *Genes only differently regulated in OE::hasA vs. WT*.

** *Genes only genes only differently regulated in OE::hasA vs. OE::hasA/ΔhasD*.

Additionally, genes from several predicted but uncharacterized clusters also are positively affected: five and eight genes from two uncharacterized NRPS-like gene clusters on chromosome III (AFUA_3g02670; 02680, 02685, 02690, and 02760) and V (AFUA_5g09960; 10040, 10090, 10160, 10180, 10200, 10210, and 10220), respectively, as well as one gene belonging to an uncharacterized PKS cluster on chromosome I (AFUA_1g17690), four genes belonging to an uncharacterized PKS gene cluster on chromosome III (AFUA_3g01400; 01460; 01500, and 01560) and three genes belonging to an uncharacterized PKS cluster on chromosome IV (AFUA_4g14530, 14570, and 14580) (Table 1). Interestingly, the uncharacterized PKS-encoding gene on chromosome I (AFUA_1g17740) as well as one of the uncharacterized PKS-encoding gene on chromosome III (AfuA_3g14700) and additional five genes from the same cluster (AFUA_3g14710, 14740, 14750; 14760, and 14770) were only found to be up-regulated comparing the OE::*hasA* strain to OE::*hasA*/Δ*hasD*, in which neither HAS nor any intermediate is produced (Table 1). Similarly, three (*fgaPT1*/*easL, fgaDH*/*easD*, and *fgaP450-2*/*easM*) of the 11 characterized genes from the fumigaclavine gene cluster (Unsold and Li, 2006; Wallwey et al., 2010; Robinson and Panaccione, 2012; Wallwey et al., 2012) as well as one uncharacterized NRPS-encoding gene [AFUA_3g13730; *nrps6*/*pesG*; (Cramer et al., 2006b; Stack et al., 2007)] were only found to be up-regulated when comparing OE::*hasA* to OE::*hasA*/Δ*hasD* (Table 1).

Of the secondary metabolite gene clusters found to be down-regulated in the common gene set, all of the 13 gliotoxin cluster genes (*gliZ, gliI, gliJ gliP, gliC, gliM, gliG, gliK, gliA, gliN, gliF, gliT*, and *gliH*) (Gardiner et al., 2005; Balibar and Walsh, 2006; Schrettl et al., 2010b; Davis et al., 2011; Forseth et al., 2011; Scharf et al., 2012a,b) (Figure 10), as well as the adjacently located NRPS-encoding gene *nrps9*/*pesJ* [AFUA_6g09610; (Cramer et al., 2006b; Stack et al., 2007)], seven (*ftmA, ftmB, ftmC, ftmD, ftmE, ftmG*, and *ftmH*) of the eight fumitremorgin cluster genes (Maiya et al., 2006; Grundmann et al., 2008; Kato et al., 2009; Steffan et al., 2009; Wiemann et al., 2013), and three (*pyr6, pyr7*, and *pyr8*) of the nine pyripyropene cluster genes (Itoh et al., 2012) were identified (Table 1). Additionally, one gene (AFUA_1g10370) adjacent to the NRPS-encoding gene *nrps1*/*pes1*/*pesB* [AFUA_1g10380; (Cramer et al., 2006b; Reeves et al., 2006; O'Hanlon et al., 2012)] and the uncharacterized PKS-encoding gene (AFUA_3g02570) were also negatively affected (Table 1).

Overall, as noted above, the effect of *hasA* overexpression was dampened in the absence of *hasD*, with the fold differences in the secondary metabolite clusters often being smaller and the number of differentially expressed genes fewer. These results suggest that for the majority of gene clusters HAS itself, and not just HasA, plays a role in regulation of secondary metabolite biosynthetic genes.

Another category that we found to be enriched specifically among the genes positively affected comparing OE::*hasA* to the WT and OE::*hasA* to OE::*hasA*/Δ*hasD* belong to the functional group “heavy metal ion transport” (Figure S1; Table S2). This included the metalloreductase-encoding gene *fre2*/*freB*, the putative ferric chelate reductase-encoding gene *fre7*, the putative zinc transporter *fetD*/*fet4*, and two putative siderophore transporters (AFUA_3g13670 and AFUA_7g04730) (Table 2) all previously identified to be induced by iron or zinc starvation (Schrettl et al., 2008; Yasmin et al., 2009; Blatzer et al., 2011a,b). Two of the genes in this category were also induced by exposure to neutrophils (Sugui et al., 2008), one of them encoding a putative high affinity copper transporter (AFUA_2g03730) and the other one encoding a putative metalloreductase (AFUA_6g02820) (Table 2). These results support the hypothesis that HAS is a component of an iron-centered regulon, that when over-produced induced iron starvation responsive genes.

**Table 2:** **Genes significantly up-regulated comparing OE::*hasA* vs. WT and OE::*hasA* vs. OE::*hasA*/Δ*hasD* of the category “heavy metal ion transport**.”

**AFUA ID**	**Gene name/Predicted function**	**log(2) value**	
		**OE::*hasA* vs. WT**	**OE::*hasA* vs. OE::*hasA*/Δ*hasD***
AFUA_1G17270	*fre2*/*freB*	1.05	1.39
AFUA_2G03730	Copper transporter	1.71	1.88
AFUA_3G13670	Siderophore transporter	1.06	1.38
AFUA_4G03940	*fre7*	2.83	2.89
AFUA_4G14640	*fetD*/*fet4*	1.21	1.12
AFUA_6G02820	Metallo reductase	1.43	1.75
AFUA_7G04730	Siderophore transporter	1.78	1.59

On the other hand, several genes repressed by iron sufficiency in an SreA-dependent manner (Schrettl et al., 2008), were only identified to be up-regulated when comparing the OE::*hasA* strain to OE::*hasA*/Δ*hasD* (Table 3). Specifically, the gene cluster located on chromosome III (AfuA_3g03390-03440) involved in siderophore production is up-regulated in this comparison. Genes identified in this cluster encode for the hydroxyornithine transacylase SidF, the fusarinine C NRPS SidD, the ABC transporter SitT and the fusarinine C acyltransferase SidG (Table 3).

**Table 3:** **Genes significantly up-regulated comparing OE::*hasA* vs. OE::*hasA*/Δ*hasD* involved in siderophore biosynthesis and SreA-repressed**.

**AFUA ID**	**Gene name/Predicted function**	**log(2) value**	
		**OE::*hasA* vs. WT**	**OE::*hasA* vs. OE::*hasA*/Δ*hasD***
AFUA_3G03390	Siderophore esterase	0.56	1.78
AFUA_3G03400	*sidF*	−0.59	2.06
AFUA_3G03410	enoyl-CoA hydratase	−0.29	2.33
AFUA_3G03420	*sidD*	0.06	1.50
AFUA_3G03430	*sidT*	0.60	1.50
AFUA_3G03440	Siderophore transporter	0.62	2.20

Several of the genes negatively affected in both comparisons (OE::*hasA* to WT and OE::*hasA* to OE::*hasA*/Δ*hasD*) belong to Fe-dependent enzymes involved in primary metabolism (Figure S2; Dataset S2), supporting the hypothesis that overproduction of HAS leads these strains to experience iron-deficiency. Among those genes were eight putative oxidoreductases (AFUA_1g17430; AFUA_2g03820; AFUA_2g09355; AFUA_3g00150; AFUA_3g01070; AFUA_4g13780; AFUA_5g09720; AFUA_6g03290) (Table 4). In line with this finding we observed an up-regulation of the cytochrome C-encoding gene (*cycA*) in northern blot analysis (Figure 5B). Similar to what was observed for the genes belonging to secondary metabolism, the global effects of *hasA* overexpression were also lessened in the absence of *hasD*. In this case, only 101 and 194 genes were up- or down-regulated in the OE*::hasA*/Δ*hasD* strain compared to WT, with less than a third of those having differences greater than four-fold, supporting our the hypothesis that HAS itself causes the major effects observed (Dataset S1).

**Table 4:** **Genes significantly down-regulated comparing comparing OE::*hasA* vs. WT and OE::*hasA* vs. OE::*hasA*/Δ*hasD***.

**AFUA ID**	**Gene name/Predicted function**	**log(2) value**	
		**OE::*hasA* vs. WT**	**OE::*hasA* vs. OE::*hasA*/Δ*hasD***
AFUA_1G17430	Putative oxidoreductase	−1.75	−1.58
AFUA_2G03820	Putative oxidoreductase	−1.68	−1.43
AFUA_2G09355	Putative oxidoreductase	−1.28	−1.10
AFUA_3G00150	Putative oxidoreductase	−1.39	−1.04
AFUA_3G01070	Putative oxidoreductase	−1.64	−1.35
AFUA_4G13780	Putative oxidoreductase	−1.60	−1.58
AFUA_5G09720	Putative oxidoreductase	−1.54	−1.27
AFUA_6G03290	Putative oxidoreductase	−2.35	−1.32

## Discussion

**Figure 11:**
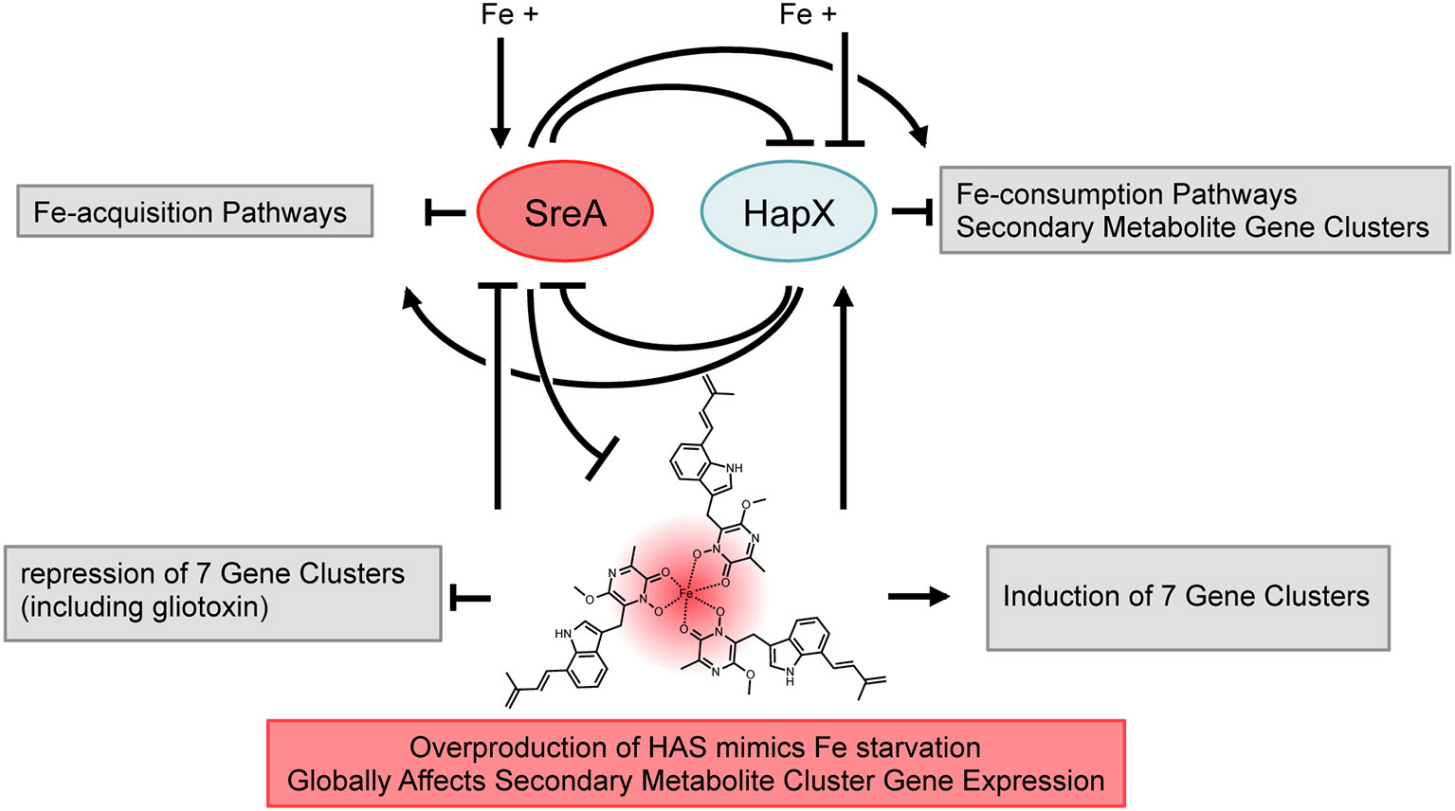
**Iron-dependent regulation in *Aspergillus fumigatus***. The two iron-dependent transcription factors SreA and HapX are in the center of an iron-dependent feed-back loop controlling iron-acquisition and -consumption pathways (Haas, 2012). Iron availability induces production of secondary metabolism, with SreA and HapX acting as activator and repressor. Production of Terezine D (HAS precursor) is repressed by SreA. Overproduction of HAS caused iron starvation conditions resulting in deregulation of iron-acquisition and -consumption pathways, most likely through SreA and HapX. Overproduction of HAS has global influence on expression of secondary metabolite gene clusters.

Initial characterization of the HAS biosynthetic pathway resulted in two significant findings, one being that the HAS bound iron(III) and another that HAS and the cluster transcription factor, *hasA*, might be involved in production of other small molecules (Yin et al., 2013). The binding with iron led to the hypothesis that HAS production might impact iron homeostasis in *A. fumigatus* whereas the latter observation spoke of a global influence on secondary metabolite production. To investigate these observations further, we coupled a genomic transcriptional profile analysis of *hasA* and *hasD* mutants with biochemical examination of these strains and iron homeostasis mutants, in the process uncovering a heretofore uncharacterized iron-responsive secondary metabolite network in *A. fumigatus*. A model of our findings is illustrated in Figure 11 where we place HAS as a component of the iron regulon that ties into global regulation of secondary metabolic clusters.

Characterization of HAS mutants on iron deficient and iron replete medium was essential for understanding the impact of this metabolite on fungal physiology. Our first query was to determine if HAS exhibited any properties of a siderophore and thus compared growth of *hasA* mutants to that of Δ*sidA*, lacking the L-ornithine-*N*^5^-monooxygenase SidA, the first committed step required for both extracellular and intracellular siderophore biosynthesis in *A. fumigatus* (Schrettl et al., 2004a). Deletion of *hasA* had no gross impact on growth on medium lacking iron and OE::*hasA* in a Δ*sidA* background did not restore growth on iron deficient media (Figure 1). However, there were several significant aberrancies in both the OE::*hasA* and the OE::*hasA*/Δ*hasG* (making the penultimate iron-binding precursor astechrome) strains grown on various concentrations of iron whereas the OE::*hasA*/Δ*hasD* strain (unable to produce a iron(III) binding molecule) demonstrated a wild type growth pattern.

First among the phenotypes in the OE::*hasA* and OE::*hasA*/Δ*hasG* strains was the accumulation of red pigmentation in low (0.03 mM) and high (1.5 mM) iron supplementation with the red intensity proportional to the iron content (Figure 2). Pigmentation was absent in the OE::*hasA*/Δ*hasD* strain. The OE::*hasA* strain also showed a statistically significant submersed growth deficit in iron concentrations below 1 mM (Figure 3) which was not obvious on agar medium (Figure 1). The concomitant increase in extracellular siderophore content in these same conditions coupled with decreased intracellular siderophore content at 0 mM iron in the OE::*hasA* strain (Figure 4) suggested that HAS might act as a one-way sink for iron, rendering it inaccessible to the fungus (Figure 11).

This hypothesis was supported by assessing expression of key genes associated with iron starvation. In addition to siderophore production, *A. fumigatus* also hosts an active reductive iron assimilation system with FtrA acting as a high affinity iron permease (Schrettl et al., 2004a). Both SidA and FtrA are activated during conditions of iron starvation (Schrettl et al., 2008, 2010a) and their expression was upregulated in the OE::*hasA* and OE::*hasA*/Δ*hasG* mutants. On the other hand, genes encoding proteins known to consume iron, such as cytochrome C (*cycA*) in the respiratory pathway, are down regulated in iron starvation conditions (Oberegger et al., 2002). Here *cycA* mRNA was down regulated in the HAS over-production strains (Figure 5A), yet another indication that excess HAS or astechrome results in iron deficiency. Furthermore, supporting this hypothesis, the GATA-type transcription factor, SreA, that acts as repressor under iron excess conditions (Schrettl et al., 2008) was significantly down-regulated in strains over-producing HAS (Figure 5B). Global RNA-seq analysis under iron-replete conditions support these findings, as genes involved in iron acquisition that are part of the SreA-regulon are positively affected when HAS is overproduced compared to a strain unable to produce HAS or any intermediate of the pathway (Figure 11; Tables 2, 3). Interestingly, the RNA-seq data not only suggest an up-regulation of genes involved in iron acquisition but also genes involved in copper and zinc homeostasis (Table 2). A relationship between iron and zinc as well as iron and copper homeostasis was previously described in *A. fumigatus*, where iron starvation caused an up-regulation of zinc transporters (Yasmin et al., 2009). Up-regulation of copper transporters under iron deficient conditions can best be explained by the reductive iron-uptake system of *A. fumigatus* which is copper dependent (Blatzer et al., 2011b).

Additionally to the regulatory effects on iron acquisition and consumption pathways caused by HAS production, our RNA-seq data implicate that production of HAS and not just the transcription factor HasA positively and negatively influences expression of specific *A. fumigatus* secondary metabolite gene clusters (Figure 11; Table 1). Interestingly, while the *has* genes were still overexpressed in OE::*hasA* compared to OE::*hasA/ΔhasD*, the fold overexpression decreased compared to OE::*hasA* vs. WT suggesting that either HAS itself or HasD had some influence on *has* expression (Table 1). Such a situation has been noted for the gliotoxin cluster in *A. fumigatus* and the bikaverin gene cluster in *F. fujikuroi*, where cluster gene expression is influenced by formation of the final product and the respective key enzymes GliP (NRPS) and Bik1 (PKS) (Cramer et al., 2006a; Wiemann et al., 2009). The observed effect of cluster cross-talk, where production of one metabolite influences the abundance of another one, was previously reported for *A. fumigatus*. Specifically, deletion of either the NRPS-encoding gene responsible for fumiquinazoline production (*nrps11*/*pesL*/*fqzC*/*fmqC*) or *nrps1*/*pes1*/*pesB*, involved in production of an uncharacterized non-ribosomal peptide, had significant influence on production of fumigaclavines and fumitremorgins, the former depleted and the latter found to be increased (O'Hanlon et al., 2012). It is interesting to note that the same trend was observed when HAS production was increased (Table 1). Cross-talk between gliotoxin and ergothioneine has also been reported for *A. fumigatus* and is suggested to be caused by response to a change in redox status of the cells rather than changed precursor pools (Gallagher et al., 2012). In line with these observations, exogenously added gliotoxin resulted in differentially expressed proteins involved in redox homeostasis (Carberry et al., 2012). A relation between iron and redox homeostasis is described in pro- and eukaryotic organisms, where iron-containing proteins function as reactive oxygen species (ROS)-sensors on the one hand (Outten and Theil, 2009; Outten and Albetel, 2013) and ROS-detoxifying enzymes on the other hand (Oberegger et al., 2000, 2001; Haas, 2012). Since HAS had a major impact on iron homeostasis, it is feasible that the effects observed on secondary metabolism are a consequence of the changes in iron availability through modification of redox homeostasis.

This hypothesis, that the global impact of HAS could be in large part mediated by iron availability, was supported by both the effect of HapX and SreA loss on secondary metabolite output as well as iron feeding impacts on small molecule production (Figures 7, 9). With the exception of fumitremorgin production, all analyzed metabolites including terezine D (the first stable intermediate in the HAS pathway) were significantly increased when ambient iron concentrations were increased from 0.03 to 1 mM, suggesting that SreA might execute activating effects under iron excess conditions (Figure 8). Supporting this hypothesis, this induction of two secondary metabolites (pseurotin A and fumagillin) was lost in the Δ*sreA* strain (Figures 7, 8) and production was significantly reduced compared to the wild type in 1 mM iron (Figure 9). However, production of terezine D was significantly increased in the Δ*sreA* strain compared to the WT in 0.03 and 1 mM iron, indicating that SreA negatively affects HAS production under these conditions, similarly to iron acquisition pathways identified by Schrettl et al. (2008) (Figure 11). Deletion of *sreA* was also shown to induce increased iron toxicity due to uncoordinated iron uptake (Schrettl et al., 2008), possibly yet another route by which *sreA* loss could affect secondary metabolism.

As predicted by our model (Figure 11), deficiency of HapX alters secondary metabolite production mainly under iron deplete conditions, where it seems to function as a repressor (Figures 6, 8, 9). HapX is part of the CCAAT-Box binding complex in filamentous fungi (Kato, 2005; Hortschansky et al., 2007), and was shown to be an inducer of siderophore production in *A. fumigatus* (Schrettl et al., 2010a) and is directly implicated to regulate the penicillin gene cluster in *A. nidulans* (Litzka et al., 1996). This work presents a role for HapX in repressing expression and subsequent production of several secondary metabolite clusters.

Summarizing our combined data, we show here that production of the iron(III)-chelating metabolite HAS is strongly regulated by the iron-induced transcription factor SreA, and HAS production feeds into regulation of *sreA* expression, thereby eliciting an iron-depletion response in iron-replete conditions. These findings suggest overexpression of HAS and deficiency of both iron-responsive elements HapX and SreA have significant effects on secondary metabolism, thus placing iron availability as one environmental trigger of secondary metabolism (Figure 11). This impact of iron on secondary metabolism could be direct (many secondary metabolite enzymes require iron as a cofactor) or through changes in primary metabolism, possibly through alterations in amino acid pools that have previously been reported to be coordinated by iron-availability in a Hapx-dependent manner in *A. fumigatus* (Schrettl et al., 2008). Precedence for primary metabolism affecting secondary metabolism comes from *F. fujikuroi*, where mutations of specific amino acids in the glutamine synthetase had significant effects on amino acid composition and secondary metabolite production (Wagner et al., 2013). It is tempting to speculate that the effects of HAS and iron gradients on secondary metabolism are a reflection not only of iron availability but of accessible primary metabolite pools and/or redox homeostasis.

### Conflict of interest statement

The authors declare that the research was conducted in the absence of any commercial or financial relationships that could be construed as a potential conflict of interest.

## Supplementary material

Figure S1 **Fold enrichment of functional categories of the common gene set at least two-fold up-regulated in OE::*hasA* compared to WT and OE::*hasA*/Δ*hasD***. Gene sets were analyzed using the Munich Information Center for Protein Sequences Functional Catalog as described in Material and Methods.

Figure S2 **Fold enrichment of functional categories of the common gene set at least two-fold down-regulated in OE::*hasA* compared to WT and OE::*hasA*/Δ*hasD***. Gene sets were analyzed using the Munich Information Center for Protein Sequences Functional Catalog as described in Material and Methods.

Figure S3 **Southern analysis of Δ*hasA*, Δ*sidA* and OE::*hasA*/Δ*sidA* strains**. Genomic DNA of indicated strains was subject to restriction enzyme digestion using either BamHI or HindIII. WT = parental wild type. For deletion of *hasA*, the WT shows the expected band of 214 bp and transformants T6 and T9 show the expected band of 6300 bp. For deletion of *sidA*, the WT shows the expected band of 4000 bp and transformants T1–5 (Δ*sidA*) and T1–4 (OE::*hasA*/Δ*sidA*) show the expected band of 2300 bp.

Table S1 **Strains used in this study**.

Table S2 **Oligonucleotides used in this study**.

Dataset S1 **Excel file containing expression levels based on normalized reads per kilobase per million mapped reads and log(2) values of WT, OE::*hasA*, and OE::*hasA*/Δ*hasD***.

Dataset S2 **Excel file containing the output of the functional enrichment analysis of the common gene set of genes at least two-fold up- and down-regulated in OE::*hasA* compared to WT and OE::*hasA*/Δ*hasD***.

Dataset S3 **Excel file containing areas under the curve for the secondary metabolites analyzed in this study as described in Material and Methods**.
