# Supplementary material for: Perturbations in small molecule synthesis uncovers an iron-responsive secondary metabolite network in Aspergillus fumigatus
Source: Front Microbiol. 2014 Oct 24;5:530. doi: 10.3389/fmicb.2014.00530 (PMC4208449; doi:10.3389/fmicb.2014.00530)
Supplement: Table S1 — Strains used in this study. [file DataSheet4.DOCX]

**Table S1: Strains used in this study**

| name | strain ID | parental strain | genotype | reference |
| --- | --- | --- | --- | --- |
| Af293 | WT |  |  | (Xue et al., 2004) |
| Af293.1 |  |  | pyrG1 | (Xue et al., 2004) |
| OE::*hasA*; pyrG1 | TWY22.2 | Af293.6 | gdpA(p)::hasA::argB; pyrG1 | (Yin et al., 2013) |
| OE::*hasA* | TWY32.1 | Af293.6 | gpdA(p)::hasA::argB; A.p.pyrG | (Yin et al., 2013) |
| ∆*hasA* | TWY38.6 | Af293.1 | ∆hasA::A.p.pyrG | this study |
| OE::*hasA*/∆*hasD* | TWY24.121 | Af293.6 | gpdA(p)::hasA::argB; ∆hasD::A.p.pyrG | (Yin et al., 2013) |
| ∆*sidA* | TWY35.1 | Af293.1 | ∆sidA::pyrG | this study |
| OE::*hasA*/∆*sidA* | TWY36.1 | Af293.6 | gpdA(p)::hasA::argB; ∆sidA::pyrG | this study |
| Af293.1 comp. | TJW55.2 |  | A.p.pyrG | (Bok and Keller, 2004) |
| ATCC46645 | WT |  |  | American type Culture Collection |
| ∆*sreA* |  | ATCC46645 | ∆sreA::hph | (Schrettl et al., 2008) |
| ∆*hapX* |  | ATCC46645 | ∆hapX::hph | (Schrettl et al., 2010) |
| CEA17 | WT |  | ∆akuB::A.f.pyrG | (da Silva Ferreira et al., 2006) |
| ∆*hasA* | TWY37.2 | CEA17 KU80∆ pyrG1 | ∆hasA::A.p.pyrG | this study |
| OE::*hasA* | TJW109.3 | CEA17 KU80∆ pyrG1 | gpdA(p)::hasA::A.p.pyrG | (Yin et al., 2013) |
| OE::*hasA*/∆*hasD* | TWY25.5 | CEA17 KU80∆ pyrG1 | gpdA(p)::hasA::A.p.pyrG; ∆hasD::hph | (Yin et al., 2013) |
| OE::*hasA*/∆*hasG* | TWY28.3 | CEA17 KU80∆ pyrG1 | gpdA(p)::hasA::A.p.pyrG; ∆hasG::hph | (Yin et al., 2013) |

**Table S2: Oligonucleotides used in this study**

| name | sequence | application |
| --- | --- | --- |
| KOhasA5F | AAGGAAGATCACCGTCAACGCG | *hasA* deletion |
| KOhasA5R | CAAGCTATCGATACCTCGACTCAGTGGTGCCAAGAGTTCTCAACCAAGGC | *hasA* deletion |
| KOhasA3F | GCAGCCTCTCCGATTGTCGAATAATAGAAATAAAATGTGGCATGACCATGGC | *hasA* deletion |
| KOhasA3R | AGATGCAGACAGTAGAAAGACCC | *hasA* deletion |
| KOsidA5F | CCAGGGTAGGTGTAGAATG | *sidA* deletion |
| KOsidA5R | ccaattcgccctatagtgagtcgtattacgGATTAGGTACCTATACTTGAGG | *sidA* deletion |
| KOsidA3F | cagcttatcgatgataagctgtcaaacatgagGGAAAAG CCGGATTGCATAG | *sidA* deletion |
| KOsidA3R | CTGTCACTAGTTCGATGTAG | *sidA* deletion |
| cycA-F | CCTTTCTTGCAGTGTCCTCG | Northern probe |
| cycA-R | GTCCGGTGAACGTGCCGCGC | Northern probe |
| sidA-F | CTACATGCGTTGGTGTGCGC | Northern probe |
| sidA-R | GGTGGCCCTGTACCGCCGC | Northern probe |
| sreA-F | CGGTTCCCATCCACTCGTGG | Northern probe |
| sreA-R | GAAGCATTTCACGCATCTCC | Northern probe |
| ftrA-R | CAGATGGTATCGTGCTGACC | Northern probe |
| ftrA-F | GACAAGAGCAAGATGCTACC | Northern probe |
|  |  |  |
