# Supplementary figures and images for: Perturbations in small molecule synthesis uncovers an iron-responsive secondary metabolite network in Aspergillus fumigatus
Source: Front Microbiol. 2014 Oct 24;5:530. doi: 10.3389/fmicb.2014.00530 (PMC4208449; doi:10.3389/fmicb.2014.00530)

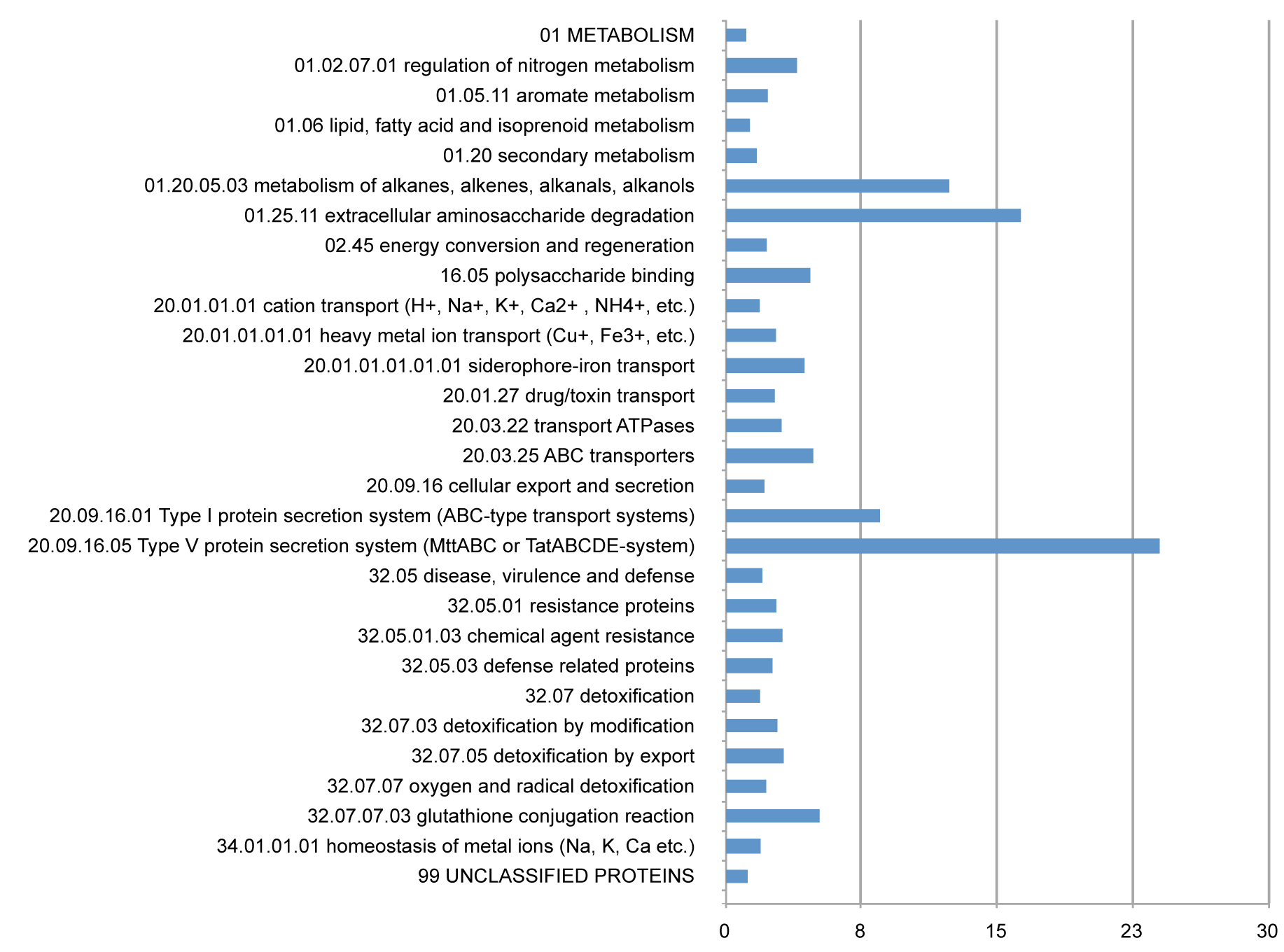

Supplement: Dataset S1 — Excel file containing expression levels based on normalized reads per kilobase per million mapped reads and log(2) values of WT, OE::hasA, and OE::hasA/ΔhasD. [file Image1.TIF]

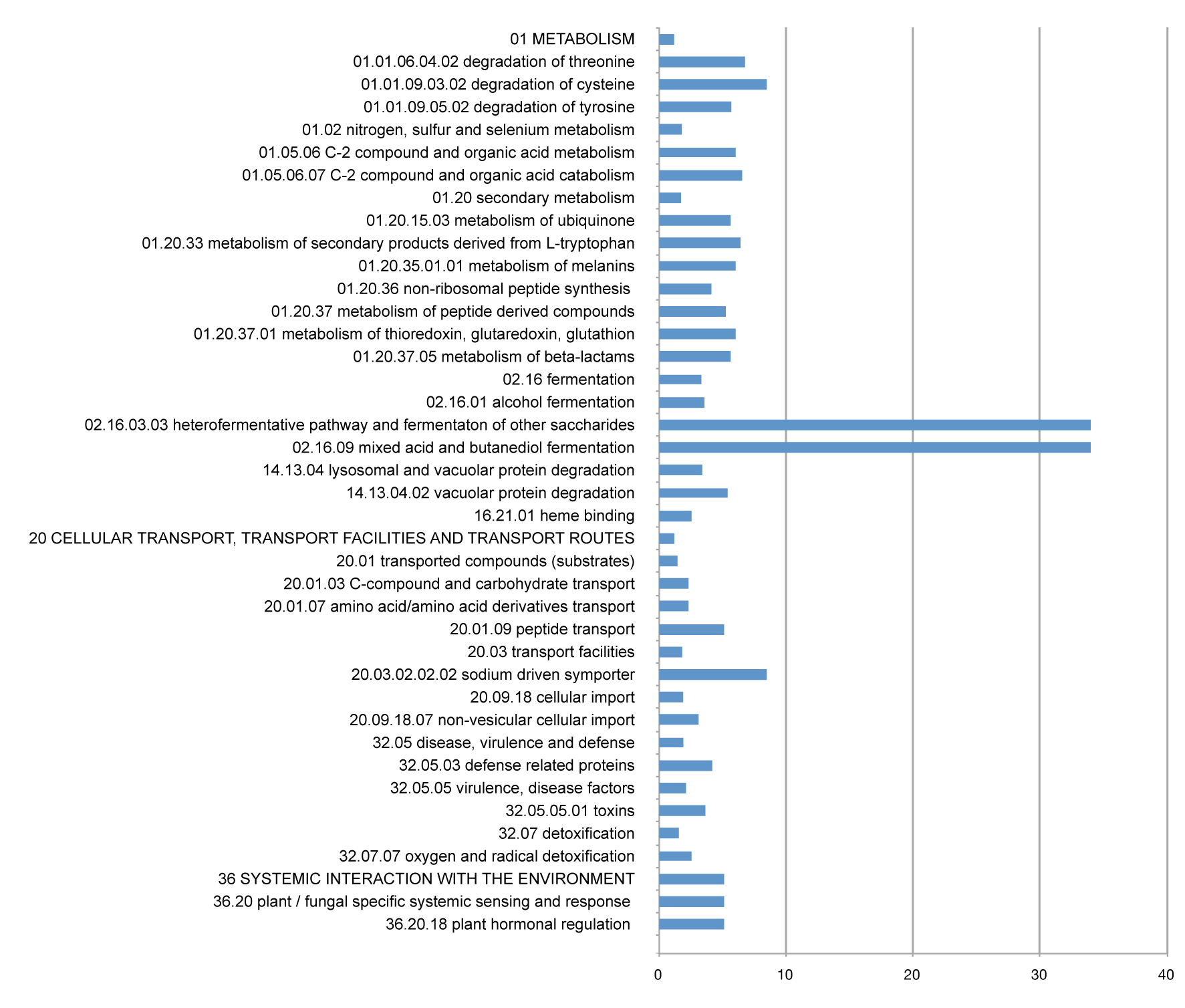

Supplement: Dataset S2 — Excel file containing the output of the functional enrichment analysis of the common gene set of genes at least two-fold up- and down-regulated in OE::hasA compared to WT and OE::hasA/ΔhasD. [file Image2.TIF]

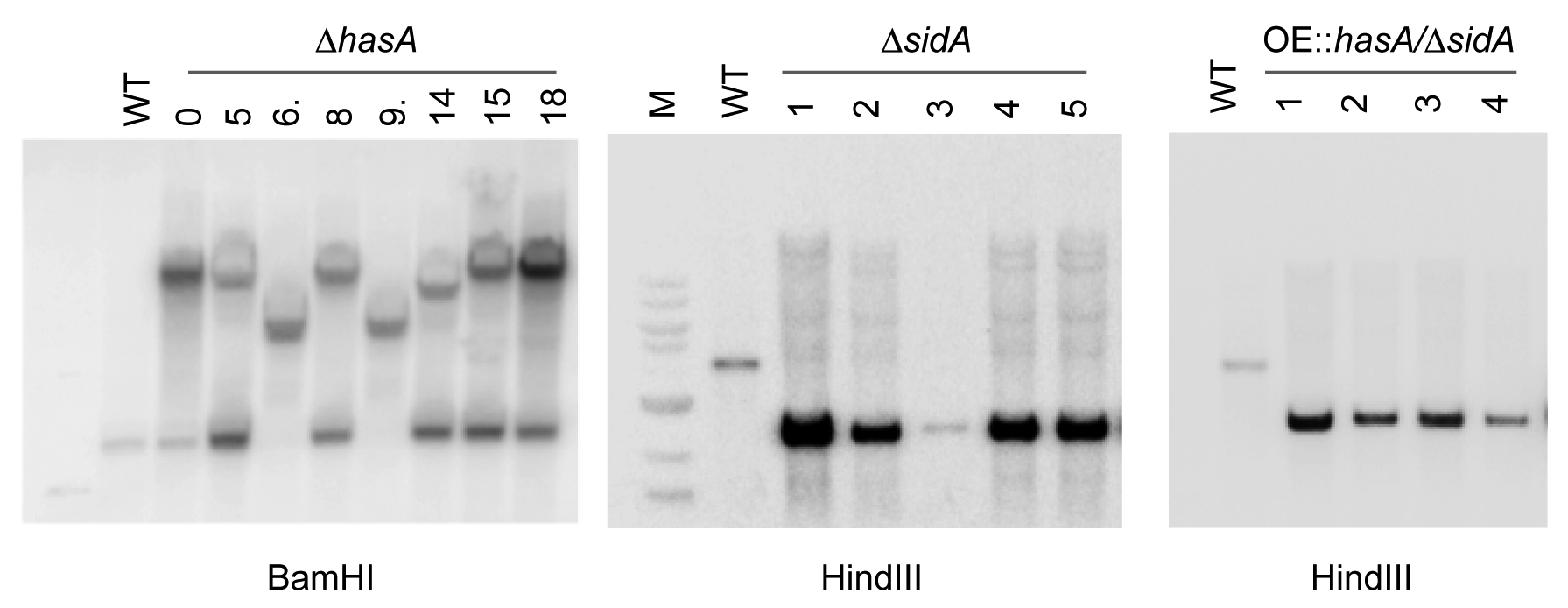

Supplement: Dataset S3 — Excel file containing areas under the curve for the secondary metabolites analyzed in this study as described in Material and Methods. [file Image3.TIF]
